# Anharmonic Vibrational Raman Optical Activity of Methyloxirane: Theory and Experiment Pushed to the Limits

**DOI:** 10.1021/acs.jpclett.2c02320

**Published:** 2022-09-20

**Authors:** Qin Yang, Josef Kapitán, Petr Bouř, Julien Bloino

**Affiliations:** # Scuola Normale Superiore di Pisa, Piazza dei Cavalieri 7, 56126 Pisa, Italy; ‡ Department of Optics, Palacký University Olomouc, 17. listopadu 12, 77146 Olomouc, Czech Republic; † Institute of Organic Chemistry and Biochemistry, Academy of Sciences, Flemingovo náměstí 2, 16610 Prague, Czech Republic

## Abstract

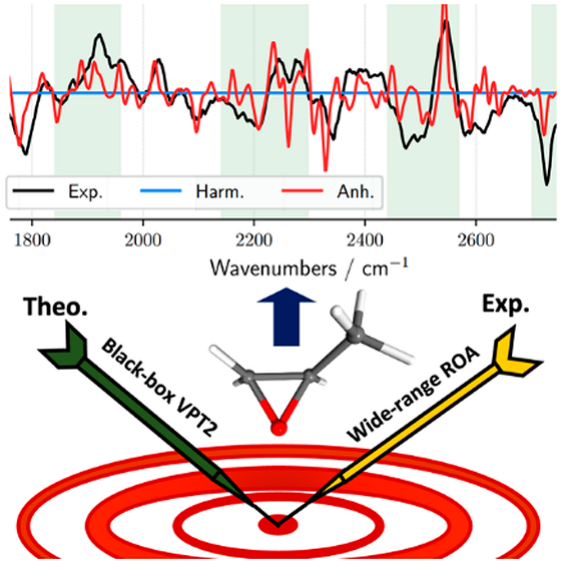

Combining Raman scattering and Raman optical activity (ROA) with computer simulations reveals fine structural and physicochemical properties of chiral molecules. Traditionally, the region of interest comprised fundamental transitions within 200–1800 cm^–1^. Only recently, nonfundamental bands could be observed as well. However, theoretical tools able to match the observed spectral features and thus assist their assignment are rather scarce. In this work, we present an accurate and simple protocol based on a three-quanta anharmonic perturbative approach that is fully fit to interpret the observed signals of methyloxirane within 150–4500 cm^–1^. An unprecedented agreement even for the low-intensity combination and overtone transitions has been achieved, showing that anharmonic Raman and ROA spectroscopies can be valuable tools to understand vibrations of chiral molecules or to calibrate computational models.

Raman optical activity (ROA) has been extensively used in recent decades to reveal unique structural and physicochemical properties of molecules.^1−3^ Because of the complexity of the signal, the interpretation of the spectra depends heavily on the theory. Together with experimental limitations, this has confined ROA studies to the fingerprint region dominated by fundamental transitions.^3,4^ Only recent works have shown that nonfundamental transitions and those outside the fingerprint region can also significantly contribute to the understanding of molecular structures and interactions.^5−7^ So far, such efforts remain scarce because of limitations of both experiment and theory. For ROA, tiny differences in Raman scattering of right and left circularly polarized light are difficult to measure. This is further exacerbated for nonfundamental transitions as they have much lower intensities than the fundamental ones.^8^ Recording their ROA, however, was made possible in this work thanks to an advanced spectrometer,^5^ and the recorded spectra of R- and S-methyloxirane (Figure 1) discussed in the present study represent one of only the few examples where nonfundamental ROA was observed. According to our knowledge, this is also the first example of an ROA spectrum covering the 3700–4500 cm^–1^ range. The “mirror image” ROA spectra of the two enantiomers confirm the quality and reliability of the measurement.

**Figure 1:**
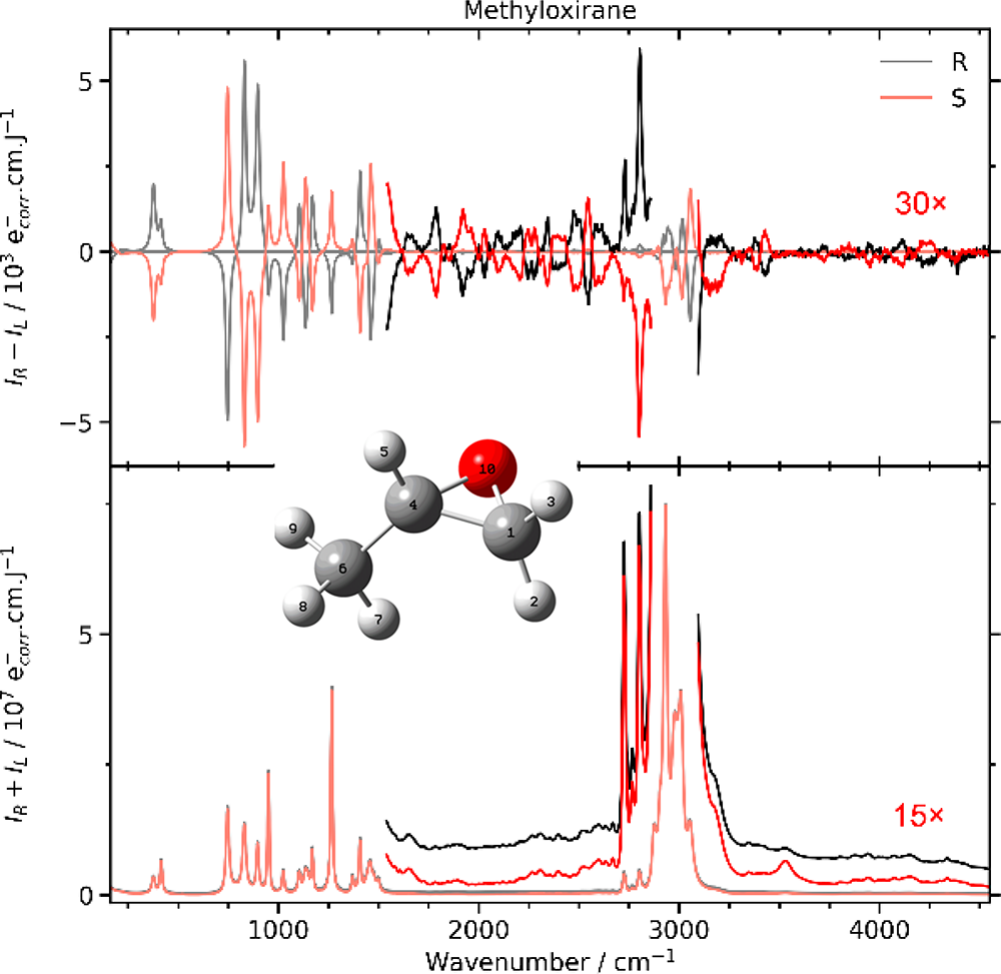
Experimental Raman (*I*_R_ + *I*_L_) and ROA (*I*_R_ – *I*_L_) spectra of R- and S-methyloxirane. Regions between 1500 and 2900 cm^–1^ and above 3100 cm^–1^ have been magnified by a factor of 30 for ROA and 15 for Raman (brighter colors) to make the fine structure visible.

To interpret all observed spectral features, an effective theoretical model is needed, which can also reproduce nonfundamental bands in a practical way. The standard harmonic approximation describes reasonably well fundamental bands but usually overestimates the vibrational energies and cannot predict the intensities of nonfundamental transitions.^8^ Indeed, the frequency energy error in the C–H stretching region can be as high as 200 cm^–1^.^9^ We have therefore used the second-order vibrational perturbation theory (VPT2), which offers a good balance between accuracy and computational cost.^8−10^ In addition, VPT2 has already proven its quality to interpret vibrational experiments.^11−15^

We had to address specific challenges. A proper treatment of resonances, near degenerations of vibrational energy levels, appeared essential. The general VPT2 (GVTP2) automatically identifying the resonances has been adopted for this purpose. The quality of the simulations obviously also depends on the reliability of the electronic structure calculation. For that, the density functional theory (DFT) is a good choice, considering its scalability to treat even larger molecular systems and its capacity to predict all properties of interest for ROA. Especially the double-hybrid functionals give accurate molecular force fields in a reasonable time.^16,17^

Methyloxirane itself is a good chiral model to calibrate experimental and theoretical methods.^6,18−21^ Despite its small size and rigid structure, its vibrational properties may be complicated because of the semifree rotating methyl group (see the structure in Figure 1). This is a type of large-amplitude vibrational motion, which sometime requires extensive variational treatments.^21−23^ We nevertheless found a limited coupling between it and other vibrational modes. Indeed, GVPT2 calculations were carried out by including or excluding the coupling, and only minor changes in the resulting spectra were observed (see Figure S1 of the Supporting Information). Therefore, it appeared more appropriate to consider the methyl group rotation in the simulations without special treatment.

First, to assess the performance of the electronic structure calculation methods without the influence of the solvent, we investigated the system in vacuum (see Figure S2 and Table S1). As noted in the literature, double hybrid functionals (B2PLYP, revDSD-PBEP86) can reach the accuracy of the “gold standard” coupled-cluster calculations with a perturbative treatment of the triple excitations, CCSD(T).^10,16^ Also in our case average errors of the vibrational energies appear similar, with double hybrid DFT performing slightly better than the standard hybrid ones. On the other hand, double hybrid calculations were about seven times longer with the same basis set. Another factor to consider is that the molecular property tensors needed for the ROA and Raman intensities are not available for double hybrid functionals. Taking into account all these aspects and following the previous studies,^10^ the combination of revDSD-PBEP86/jun-cc-pVTZ for the harmonic force field and B3PW91/jun-cc-pVTZ for higher-order energy derivatives and polarizabilities represents a good compromise between accuracy and computational effort, and thus, it was used here.

The compounds were measured as neat liquids. Therefore, a polarizable continuum model (PCM) based on the integral equation formalism^24,25^ was used to simulate the solvent effects in the computations. Experimentally, the main features in the vacuum spectra are conserved in the liquid.^21^ Variation of the solvent permittivity also does not change much the calculated frequencies and intensities, although exceptionally a different ROA sign is obtained than in vacuum (Figure S3). Although the approximations^26^ related to PCM and the definition of the cavity might lead to numerical instabilities, we did not observe such problems when calculating the anharmonic parameters. The step used in the numerical differentiation (0.01 amu^1/2^ Å in normal mode coordinates) did not cause significant change in the cavity shape, which could lead to some error in the anharmonic constants. Indeed, it is possible to assess with a high level of confidence the stability of the numerical derivatives thanks to the redundancy of some quantities. Some nondiagonal constants, like the cubic force constants (e.g., f_*ijk*_ = f_*jik*_, etc.) are computed multiple times by symmetry, i.e., displacement along each normal coordinate involved in the constant. It is possible to confirm that the cavity is stable, but also that a true minimum is reached, by comparing the numerical values of the duplicate constants. To account for the limits of numeric precision, a variation below 1 cm^–1^ was considered as negligible.

For a complete account of the anharmonicity in the transition probabilities necessary for the intensities, two elements must be considered: the wave function, responsible for the so-called mechanical anharmonicity, and the property. The latter is sometimes referred to as the electrical anharmonicity in reference to the electric dipole. The calculations involve Taylor expansions of each quantity in nuclear coordinates about the equilibrium geometry. To match the expansion of the potential energy needed at the VPT2 level, all polarizabilities involved in ROA, that is, the electric dipole-electric dipole (α), the electric dipole-magnetic dipole (*G*′), and electric dipole-electric quadrupole (*A*) tensors, need to be expanded up to the third order. It is noteworthy that, in numerical differentiations, each point is independent of the others and the computations can be fully done in parallel. The band shapes obtained by considering the mechanical (force field) and property-related (α, *A*, and *G*) anharmonicity are compared in Figure S4. The anharmonic corrections have clearly a limited impact on the strongest fundamental bands in the fingerprint and C–H stretching regions (Figure S4, upper panel). However, none of them alone can reproduce the combinations and overtones; only joint inclusion is adequate.

Another interesting fundamental aspect that has been rarely addressed but that can be conveniently tested on methyloxirane ROA is the importance of the Coriolis effects for spectral intensities. This effect stems from the coupling between vibrations and rotations, even when the Eckart conditions are satisfied, and is normally neglected at the harmonic level. Its importance within the total anharmonic correction is illustrated in Figure S5. As can be seen, Coriolis effects do not have a big impact on the Raman or ROA spectra of methyloxirane. The fundamental frequencies and intensities are virtually unchanged, and only occasionally are Raman and ROA intensities of the anharmonic transitions affected.

Raman and ROA spectra calculated with and without the anharmonic corrections are compared to experiment in Figure S6. Figure 2 shows the most intense bands, related to fundamental transitions, occurring in zones marked I (fingerprint region, 150–1700 cm^–1^) and III (C–H stretching region, 2900–3290 cm^–1^). Lower-intensity overtones and combination bands dominate the other two zones, II (1700–2900 cm^–1^) and IV (>3290 cm^–1^), virtually invisible at this scale. To analyze the spectra, each zone was further divided into regions labeled with Arabic numbers.

**Figure 2:**
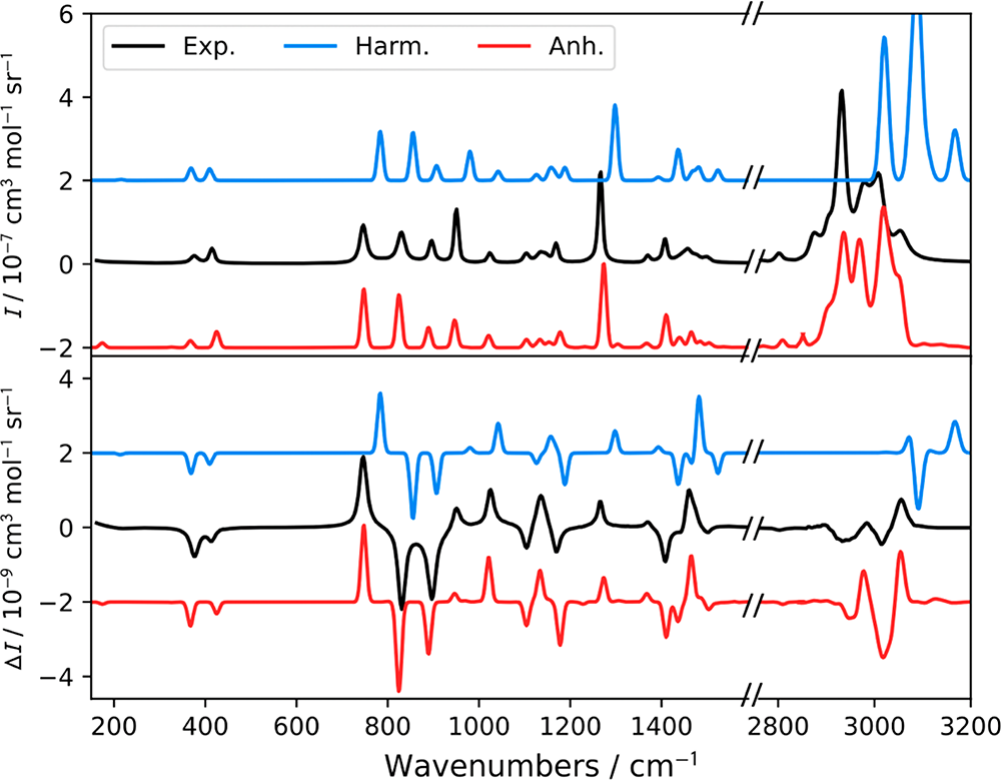
Simulated and experimental spectra of R-methyloxirane in the regions of fundamental vibrations.

Zone I is divided into I-1, I-2, I-3, and I-4 (Figure S6), with the most intense transitions listed in Table S2. Figures S7 and S8 confirms the accuracy of our simulation by reporting the errors in fundamental energies from harmonic and anharmonic simulations with respect to experimental values. I-1 is characterized by two bands around 400 cm^–1^, related to the fundamentals of modes 2 and 3. Calculations show that the two states are coupled, leading to a redistribution of the intensity compared to the harmonic level for Raman, improving the agreement with experiment. The experimental Raman intensity at 425 cm^–1^ is twice that at 369 cm^–1^, and the anharmonic simulation reproduces this ratio, while the harmonic intensities are about equal. Similar intensity redistribution and improved agreement also happens in I-4. For example, the harmonic ROA relative intensity of mode 18 is strongly overestimated, while GVPT2 predicts a mixing with the first overtone of mode 4, which lowers the intensity of the former, as observed experimentally. I-2 and I-3 are dominated by fundamental transitions from modes 4 to 13, with a less extensive state mixing, but also here the improvement brought by GVPT2 is obvious in terms of both intensities and energies.

In the CH stretching region (zone III, Figure S9), the harmonic model gives rather unrealistic intensities and a large energy error. GVTP2 results are much better, although some visible inconsistencies in intensity remain. Three regions are analyzed in detail in Table S3. III-1 is characterized by a negative ROA band and intense Raman signal, caused by 2 harmonic and 11 anharmonic transitions (although a clear distinction is problematic here). The harmonic transitions of III-1 are the 19th and 20th fundamental with a positive ROA signal. Mode 20 is coupled to the double-excited state |17(1)18(1)⟩ through a Fermi resonance, causing a transfer of intensity to the latter, which produces a negative ROA band in agreement with experiment. Modes 21 and 22 are nearly degenerate at the harmonic level but split because of the anharmonic coupling. The ROA intensity of mode 21 is then lowered and contributes to the broad feature in III-1. Mode 22 mostly impacts III-2, characterized by two wide Raman bands and a positive–negative ROA pattern. At the harmonic level, modes 21 and 22 give a single, narrow band in III-2. Inclusion of the anharmonic effects leads to a more accurate result. Interestingly, 3-quanta transitions play a significant role in modulating the negative ROA and broad Raman bands. GVPT2 thus provides the correct ROA sign pattern in III-1 and III-2. III-3 contains a positive intense ROA and a weaker Raman band, assigned to mode 24 at the harmonic level. The anharmonic calculations improve its position and relative Raman and ROA intensities. Visual discrepancies between simulation and experiment seem to be bigger for GVPT2 on Raman than on ROA. The flexibility of the methyl group is a possible cause. The positions and intensities of the Raman and ROA bands appear significantly affected by the rotation of that moiety, except for the one related to mode 24. This can be explained by the fact that the latter vibration does not involve the methyl group (Table S4).

Zones II (Figure 3) and IV (Figure 4) would normally be termed silent regions, as signals of the transitions therein are about 2 orders of magnitude smaller than those in the fingerprint or C–H stretching regions. The harmonic spectra are presented by the *y* = 0 line in Figures 3 and 4. The GVPT2 method can predict the main Raman and ROA features observed here as well. The most intense transitions are listed in Tables S5 and S6. As indicated in Figure 3, zone II was also divided into eight regions in Table S5. As an example of the predictive power of GVPT2, we can assign the predominantly positive ROA band in II-4 to combinations |15(1)5(1)⟩, |18(1)4(1) ⟩, |11(1)9(1)⟩, and |11(1)10(1)⟩, while the dip at 2262 cm^–1^ is mainly due to overtone |10(2)⟩.

**Figure 3:**
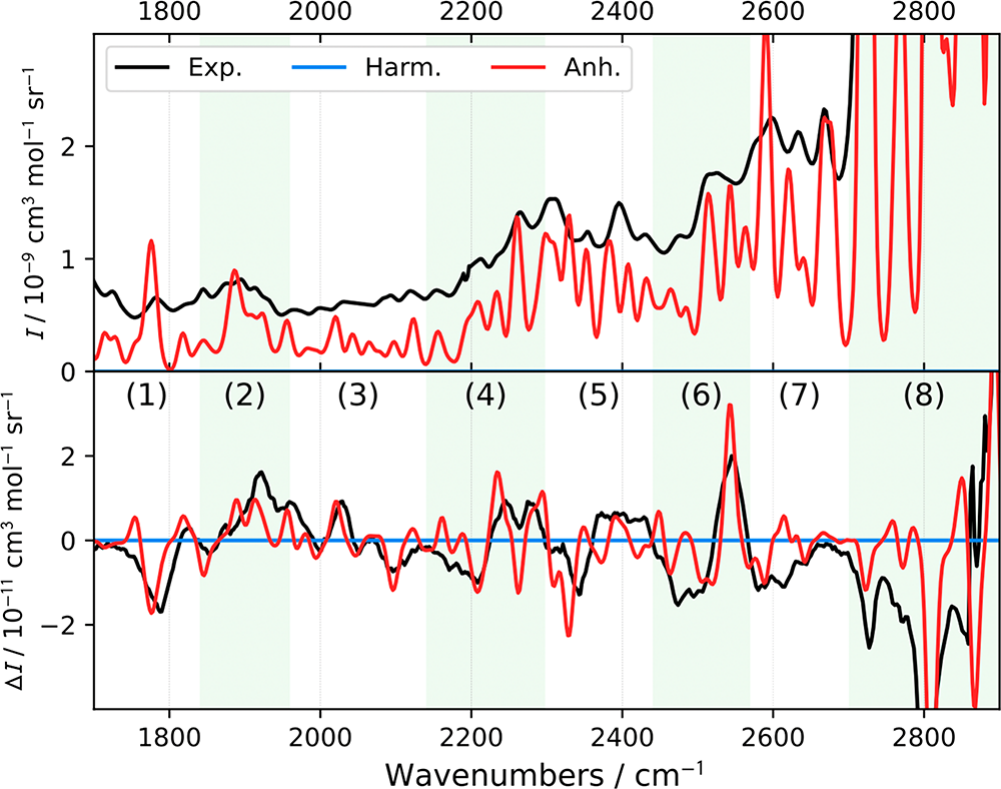
Simulated and experimental spectra of R-methyloxirane in the region of 1700–2900 cm^–1^.

**Figure 4:**
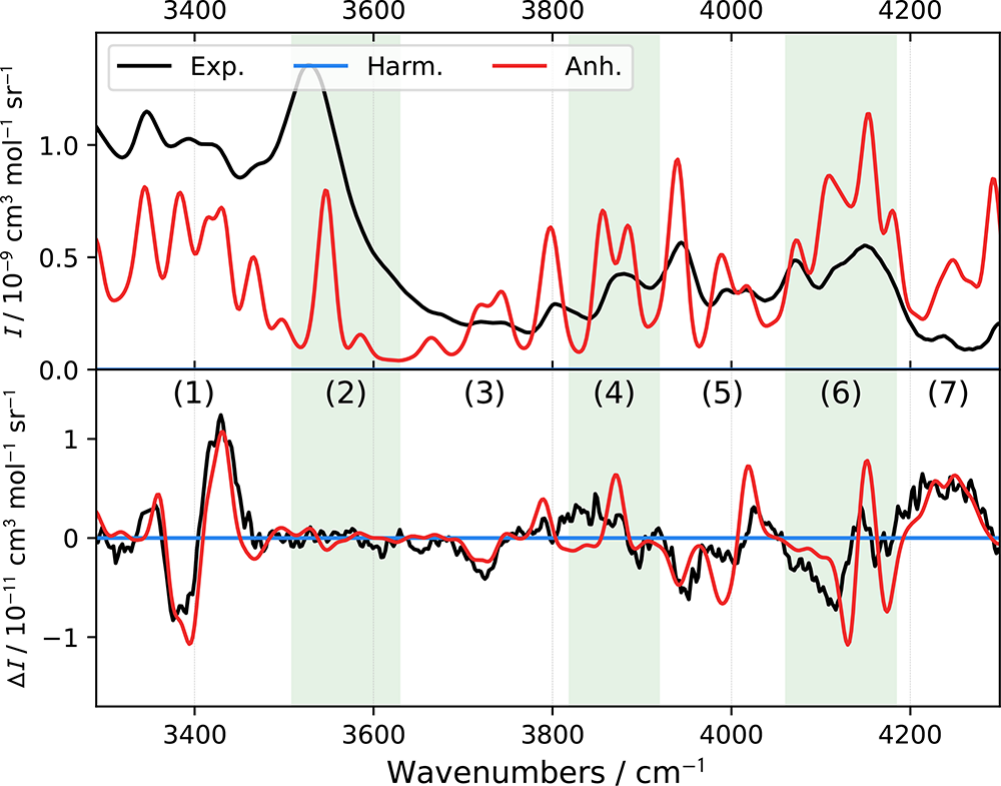
Simulated and experimental spectra of R-methyloxirane within 3290–4300 cm^–1^.

To the best of our knowledge, the ROA spectrum beyond 3300 cm^–1^ (zone IV) has never been studied or reported because of the low experimental sensitivity and lack of reliable theoretical tools needed for the interpretation. From Figure 4 and Table S6, nevertheless, the agreement between theory and experiment is still remarkable. For the analysis, seven subzones were established. The simulation matches experimental ROA better than Raman, which may be more affected by the fluorescent background. The simulated spectra thus help sort out the Raman bands coming from the studied compound, and the ROA spectra with the additional sign information provide indispensable means to verify the assignment.

We can conclude that, using the sensitive wide-range ROA spectrometer, we can capture high-quality Raman and ROA methyloxirane spectra including overtone and combination bands. The data provide a convenient reference to tune and verify the GVPT2 method as a universal tool for predicting molecular vibrational properties. In particular, we were able to test and validate criteria for the automatic resonance detection and their correction, resulting in a three-quanta GVPT2 approach capable of accurately predicting the Raman and ROA spectra in an automated way. This is a promising step in the construction of black-box protocols to predict spectra at the anharmonic level within VPT2, with an automatic identification and correction of resonances. The comparison of measured and calculated spectra provided a solid foundation for a reciprocal verification between experiment and theory, especially for the region of overtones and combinations (zones II and IV). We realize that more complex molecules may bring additional challenges to the used methodology. Inclusion of finer vibrational effects would require a further increase of precision of DFT and other electronic methods. Nevertheless, we view methyloxirane as an important step on the way to understand the vibrational behavior of molecules. It documents the power of chiroptical spectroscopy and contemporary computational chemistry.

## Supporting Information Available

Experimental and theoretical methods (PDF)
Transparent Peer Review report available (PDF)
