# Supplementary material for: Anharmonic Vibrational Raman Optical Activity of Methyloxirane: Theory and Experiment Pushed to the Limits
Source: J Phys Chem Lett. 2022 Sep 20;13(38):8888–92. doi: 10.1021/acs.jpclett.2c02320 (PMC9531246; doi:10.1021/acs.jpclett.2c02320)
Supplement: Supplementary file 1 — jz2c02320_si_001.pdf [file jz2c02320_si_001.pdf]

Anharmonic Vibrational Raman Optical Activity of Methyloxirane: Theory and Experiment Pushed to the Limits

Qin Yang,[#*] Josef Kapitán,[‡*] Petr Bouř,[†*] Julien Bloino[#*]

[#] Scuola Normale Superiore di Pisa, Piazza dei Cavalieri 7, 56126 Pisa, Italy

[‡] Department of Optics, Palacký University Olomouc, 17. listopadu 12, 77146, Olomouc, Czech Republic

[†] Institute of Organic Chemistry and Biochemistry, Academy of Sciences, Flemingovo náměstí 2, 16610, Prague, Czech Republic

Contents

Experimental methodologies

Theoretical methodologies

Table S1. Used combinations of electronic structure calculation methods (ESCMs).
Table S2. Transitions within 150-1700 cm^{-1}.
Table S3. Transitions within 2900-3290 cm^{-1}.
Table S4. Harmonic vibrational modes, corresponding anharmonic states.
Table S5. Transitions within 1700-2900 cm^{-1}.
Table S6. Transitions within 3290-4300 cm^{-1}.
Figure S1. Anharmonic spectra calculated with and without the methyl torsion.
Figure S2. Mean (MAE) and maximum (|MAX|) absolute errors for ESCMs.
Figure S3. Solvent effects.
Figure S4. Effect of properties-related and mechanical anharmonicities.
Figure S5. Influence of the Coriolis couplings.
Figure S6. Simulated and experimental spectra of R-methyloxirane in the 150-4300 cm-1.
Figure S7. Simulated and experimental spectra of R-methyloxirane within 150-1700 cm^{-1}.
Figure S8. Anharmonic and harmonic errors for the modes 1-18.
Figure S9. Simulated and experimental spectra of R-methyloxirane within 2700-3290 cm^{-1}.
Figure S10. Duschinky matrix and shift vector between B3PW91 and revDSD-PBEP86.
Figure S11. Influence of the threshold used to identify 1-1 DDRs on the band-shape.

Experimental Methodologies

Samples were purchased from Merck (S-methyloxirane: 540021-5G, R-methyloxirane: 540048-5G) and used without further purification. Raman and ROA spectra were measured by a ROA spectrometer developed at Palacký University Olomouc in collaboration with the ZEBR and Meopta companies.[1] The instrument is based on W. Hug's design using the back-scattering geometry, scattered circular polarization (SCP) modulation scheme, and diode pumped solid state laser with 532 nm excitation wavelength (Opus, LaserQuantum).[2, 3] Samples were measured in a rectangular fused silica cell of 70 μL volume (3 mm width, 4 mm depth, Starna) in the temperature-stabilized compartment at 6.0 ± 0.1°C ensuring lower evaporation of the highly volatile liquid. Raman and ROA spectra were simultaneously collected in the full range from 50 to 4560 cm^{-1}. The spectral resolution was ~ 6–8 cm^{-1}. Raman and ROA intensities were calibrated with a black-body type (tungsten-halogen) calibration source, and given as a corrected number of detected electrons per excitation energy per unit wavenumber (i.e. $e^{-}\cdot cm\cdot J^{-1}$).

The intensities of the combinational transitions are almost two orders of magnitude smaller than the intensities of the fundamental transitions. The recording of the spectra over the entire spectral range is limited by the dynamic range of the detector (pixel-well depth: readout noise ratio), where saturation is limiting for high-intensity bands and readout noise is limiting for low-intensity bands. Therefore, ROA spectra were combined from two measurements differing mainly in the exposure times of the CCD cameras: the first measurements had exposure time of 0.15 s and total accumulation time of 1.0 hour (S-enantiomer) and 2.8 hours (R-enantiomer) at a laser power of 340 mW at sample, and the second measurements had exposure time of 2.9 s and accumulation time of 18.8 h (S-enantiomer) and 32.9 h (R-enantiomer) at a laser power of 540 mW at sample. The first measurement with a short exposure time was used for spectral regions of high Raman intensities (50–160, 355–440, 703–1490, 2860–3094 cm^{-1}) and the second one for the rest of the spectrum. Please note that only ROA spectra were combined in this way – shorter accumulation Raman spectra do not have any visible noise and were used for the whole spectral range.

No background correction was applied to the ROA spectra. A fluorescent background is much more difficult to correct in the Raman signal, especially in low-intensity spectral regions.

Fortunately, the S-enantiomer sample has very small fluorescent background (noticeably smaller fluorescent background than the R-enantiomer) and only mild background correction was applied.

Theoretical Methodologies

All quantum-chemical calculations were carried out with a locally modified version of GAUSSIAN 16.[4] We used density functional theory (DFT) with hybrid (B3LYP, B3PW91) and double hybrid (revDSD-PBEP86, B2PLYP) functionals, with the jun-cc-pVTZ basis set, except where specified otherwise. Harmonic frequencies were also calculated at the CCSD(T) level. All employed electronic structure calculation methods are listed in Table S1. A "verytight" criterion was used for geometry optimizations, with maximum forces of 2×10^{-6} Hartrees/Bohr, and maximum displacements of 6×10^{-6} Å. Then, analytical harmonic force fields and vibrational frequencies were computed. Anharmonic force constants (third and fourth derivatives of the potential energy) were calculated by numerical differentiation of the second energy derivatives. In the same way, higher property derivatives were obtained numerically from analytical first derivatives. The differentiation step along the mass-weighted normal coordinates was 0.01 amu$^{1/2}$ Å.[5] The computation of the anharmonic constants is more expensive, since it requires in theory running $2N+1$ times the calculations necessary to obtain the vibrational energies and intensities at the harmonic level, with N is the number of normal modes. As the displacements are independent of one another, this process can be sped up by running each job on separate machines and carry out the numerical differentiation afterwards. The gain in time will thus depend on the number of available computing nodes. Another way to cut the computational time is to combine different levels of theory for the harmonic and anharmonic data, using a cheaper level for the latter. The principle is that the harmonic level is responsible for the largest contribution to the energies (and intensities), and the anharmonic correction is significantly smaller, so that small inaccuracies in the latter will have a marginal impact. Nevertheless, such hybrid scheme can only be applied if the two levels of theory are consistent, which means that the equilibrium geometry and the normal coordinates in the two levels ($\boldsymbol{Q}^{H}$, $\boldsymbol{Q}^{A}$) must be very close to each other. This can be checked automatically by computing a transformation like the one proposed by Duschinsky[6],

$$\boldsymbol{Q}^{\mathrm{H}} = \mathbf{J}\boldsymbol{Q}^{\mathrm{A}} + \boldsymbol{K} \qquad (1)$$

where the squared elements of the transformation matrix **J** must be above 0.9, while the elements of

the shift vector $\boldsymbol{K}$ must have a magnitude of a few dozen atomic units at most. Further technical details can be found in references.[7, 8] In this case, both J matrix and K vector present the hybrid are shown in Figure S10. Since ROA properties are not available for double-hybrid functionals, only hybrid functionals were used to compute the intensities.

In the generalized VPT2 (GVPT2) scheme, terms identified as resonant are removed from the VPT2 equations and reintroduced variationally. For the energy, this is done by diagonalizing a matrix containing the non-resonant VPT2 energies on the diagonal, and the off-diagonal couplings terms. The eigenvectors from the diagonalization are used to project the transition integrals of the desired properties obtained by discarding resonant terms onto the final, GVPT2 states.[7, 9] The identification of resonances is done automatically. To speed up the process and avoid false positives, the procedure for each type of resonance is decomposed in at least two steps. First, states close in energy are selected, then other criteria are applied. For Fermi resonances, the test proposed by Martin and coworkers was used.[10] Darling-Dennison terms do not participate in the VPT2 energies, but act as higher-order corrections. Some of them describe couplings between states that may be in resonance for the intensities and must be properly treated in the calculation of the anharmonic transition moments. For this reason, one or two tests are carried out after the states close in energies have been identified. With reference to the energy, the coupling term is simply computed. If its magnitude is above a chosen threshold, the term is included directly in the variational correction. For Darling-Dennison terms related to resonances in intensity, namely 1-1 (fundamental-fundamental coupling) and 1-3 (fundamental-three quanta state coupling), an additional test is carried out, where the coupling term is weighted by the inverse of the square of the energy difference between the coupled states. More details on the procedure can be found in Ref.[7] The earlier study had highlighted a possible shortcoming in the identification of 1-1 Darling-Dennison resonances. For this reason, we studied the influence of the criterion used to identify those resonances on the band-shape (Figure. S11). In this case, the influence of 1-1 Darling-Dennison resonance was rather weak. As expected, the impact on regions devoid of fundamental bands is null.

Based on these results, the following criteria were used. For Fermi resonances,

$$\left|\omega_i - \left(\omega_j + \omega_k\right)\right| \leq 200\text{cm}^{-1} \tag{2}$$

$$\frac{f_{ijk}^4}{(1+\delta_{jk})^2|\omega_i-(\omega_j+\omega_k)|^3} \geq 1\text{cm}^{-1} \tag{3}$$

For 1-1 Darling-Dennison resonances,

$$|\omega_i-\omega_j| \leq 100\text{cm}^{-1} \tag{4}$$

$$|\langle 1_i|\tilde{H}|1_j\rangle| \geq 10\text{cm}^{-1} \tag{5}$$

$$\frac{|\langle 1_i|\tilde{H}|1_j\rangle|}{(\omega_i-\omega_j)^2} \geq 1\text{cm} \tag{6}$$

for 2-2 Darling-Dennison resonances (between 1st overtones or 2-quanta binary combinations),

$$|\omega_i+\omega_j-(\omega_k+\omega_l)| \leq 100\text{cm}^{-1} \tag{7}$$

$$|\langle 1_i 1_j|\tilde{H}|1_k 1_l\rangle| \geq 10\text{cm}^{-1} \tag{8}$$

And for 1-3 Darling-Dennison resonances,

$$|\omega_i-(\omega_j+\omega_k+\omega_l)| \leq 100\text{cm}^{-1} \tag{9}$$

$$|\langle 1_i|\tilde{H}|1_j 1_k 1_l\rangle| \geq 10\text{cm}^{-1} \tag{10}$$

$$\frac{|\langle 1_i|\tilde{H}|1_j 1_k 1_l\rangle|}{[\omega_i-(\omega_j+\omega_k+\omega_l)]^2} \geq 1\text{cm} \tag{11}$$

where *i, j, k, l* refer to the normal modes, ω_i are the harmonic wavenumbers, f_{ijk} is the third derivative of the potential energy with respect to the dimensionless normal coordinates, q_i, q_j and q_k. $\tilde{H}$ is the contact-transformed Hamiltonian[11] and δ is the Kronecker symbol. Here, the Dirac notation was used to represent the harmonic states as a vector of number of quanta. For the sake of readability, only the non-null quanta are listed. In the rest of this document, as well as in the main manuscript, a slightly different, more intuitive form is used, where the number of quanta is indicated between parentheses, following the mode (ex: |i(1)j(2)⟩).

In the final spectra, idealized experimental spectra averaged over the two enantiomers [$\Delta I_{ROA}=(\Delta I_R-\Delta I_S)/2$ for ROA, and $I_{Raman}=(I_R+I_S)/2$ for Raman] are presented. The calculated intensities are broadened using Gaussian bands with half-widths at half-maximum (HWHM) of 7 cm^{-1} below 2900 cm^{-1} and 10 cm^{-1} above 2900 cm^{-1}. The simulated spectra in the range of 2900~4500 cm^{-1} are shifted by -18 cm^{-1}.

Table S1 Used combinations of electronic structure calculation methods (ESCMs). B2PLYP[12] and revDSD-PBEP86[13] are double-hybrid DFT functionals.[14] B3LYP,[15] PW6B95,[16] B3PW91[15] are hybrid functionals. Empirical dispersion (D3)[17] was employed for all functionals, together with Becke-Johnson damping (BJ)[17]; for B2PLYP, only the former was used. In addition to Dunning's aug-cc-pVTZ[18, 19], two types of Truhlar's "calendar" basis set have been used (jun-cc-pVTZ, jul-cc-pVTZ). [19]

No.	Combinations	Abbreviations
**1**	B2PLYP-D3/aug-cc-pVTZ	B2PD/ATZ
**2**	B2PLYP-D3/jun-cc-pVTZ	B2PD/JNTZ
**3**	revDSD-PBEP86-D3BJ/jun-cc-pVTZ	RDSD/JNTZ
**4**	B3LYP-D3BJ/jun-cc-pVTZ	B3LD/JNTZ
**5**	PW6B95-D3BJ/jun-cc-pVTZ	PW6D/JNTZ
**6**	B3PW91-D3BJ/jun-cc-pVTZ	B3PD/JNTZ
**7**	B3PW91-D3BJ/jul-cc-pVTZ	B3PD/JLTZ
**8**	CCSD(T)/aug-cc-pVTZ	CCSD/ATZ

Table S2. Transitions within 150-1700 cm^{-1}. Energy E is given in cm^{-1}, ROA and Raman intensities in 10^{-5} and 10^{-1} Å^6. i denotes the harmonic modes.

**Experiment**		**Harmonic**				**Anharmonic**			
Range	Exp.	i	E	I_{ROA}	I_{RAMAN}	States	E	I_{ROA}	I_{RAMAN}
**I-1 150- 600**	167[a]	**1**	213	-0.61	0.16	+1.00*|1(1)⟩	174	-0.85	0.67
	376	**2**	369	-7.33	1.77	+0.91*|2(1)⟩ + 0.42*|3(1)⟩	367	-8.91	1.03
	415	**3**	409	-4.02	1.68	+0.91*|3(1)⟩ - 0.42*|2(1)⟩	425	-4.39	2.42
**I-2 600-1065**	746	**4**	783	23.45	7.77	+1.00*|4(1)⟩	747	30.92	9.47
	831	**5**	855	-26.19	7.71	+1.00*|5(1)⟩	825	-36.72	8.66
	896	**6**	907	-16.36	2.38	+1.00*|6(1)⟩	889	-21.67	3.36
	949	**7**	980	2.15	4.77	+0.99*|7(1)⟩	947	3.53	4.64
	1025	**8**	1042	12.21	1.52	+1.00*|8(1)⟩	1021	19.16	2.08
**I-3 1065-1340**	1104	**9**	1126	-4.39	1.01	+0.99*|9(1)⟩	1104	-10.26	1.43
	1135	**10**	1156	6.30	1.88	+0.99*|10(1)⟩	1134	13.65	1.39
	1147	**11**	1166	2.60	1.15	+0.98*|11(1)⟩	1153	1.03	0.89
	1170	**12**	1188	-13.58	2.23	+0.99*|12(1)⟩	1178	-19.12	2.75
	1266	**13**	1298	9.64	13.35	+1.00*|13(1)⟩	1273	10.92	15.29
**I-4 1340-1700**	1369	**14**	1393	2.63	0.60	+0.99*|14(1)⟩	1368	3.78	0.75
	1408	**15**	1437	-14.33	5.67	+0.97*|15(1)⟩	1410	-16.40	6.10
	1440	**16**	1468	-5.59	1.60	+0.76*|6(1),2(1),1(1)⟩-0.63*|16(1)⟩	1436	-7.10	0.85
						+0.72*|16(1)⟩+0.63*|6(1),2(1),1(1)⟩	1443	-0.52	1.15
	1462	**17**	1482	26.14	2.38	+0.91*|17(1)⟩	1465	21.91	2.97
	1500	**18**	1524	-9.44	1.95	+0.75*|4(2)⟩+0.60*|18(1)⟩	1485	2.19	1.02
						+0.73*|18(1)⟩-0.64*|4(2)⟩	1503	-4.11	0.86

[a] *Uncertainty of ±5cm^{-1}, due to the flattening pattern of the observed spectra.*

Table S3. Transitions within 2900-3290 cm^{-1}. Symbols are the same as in Table S2.

Experimental Range	Harmonic				Anharmonic			
	i	E.	I_{ROA}	I_{RAMAN}	States	E.[a]	I_{ROA}	I_{RAMAN}
**III-1 2900-2973**	**20**	3096	28.67	32.44	+0.88*|16(1),4(2)⟩	2913	-0.03	2.77
					+0.52*|20(1)⟩ + 0.48*|18(1),17(1)⟩	2919	-3.18	9.81
	**19**	3039	0.60	55.10	+0.68*|17(1),4(2)⟩ + 0.47*|19(1)⟩ - 0.44*|17(2)⟩	2933	0.11	20.77
					+0.64*|17(1),4(2)⟩ - 0.35*|19(1)⟩	2937	2.63	9.34
					+0.81*|16(1),9(1),3(1)⟩ +0.36*|19(1)⟩	2941	1.44	11.53
					+0.74*|18(1),17(1)⟩	2945	-19.34	5.64
					+0.89*|18(1),9(1),2(1)⟩	2952	2.85	1.14
					+0.91*|16(1),11(1),2(1)⟩	2954	0.30	1.13
					+1.00*|15(1),11(1),3(1)⟩	2959	0.04	1.15
	**21**	3107	-46.07	23.64	+0.90*|21(1)⟩ - 0.35*|17(1),9(1),3(1)⟩	2964	-22.04	20.88
					+0.92*|17(1),9(1),3(1)⟩	2967	-3.81	5.06
**III-2 2973-3040**	**22**	3109	-18.38	46.30	+0.72*|22(1)⟩ -0.45*|23(1)⟩	2975	40.58	20.37
					+1.00*|16(1),12(1),2(1)⟩	2976	0.07	1.97
					+1.00*|18(1),9(1),3(1)⟩	2989	-3.57	1.95
	**23**	3128	3.38	9.78	+0.75*|16(1),15(1),1(1)⟩+0.36*|22(1)⟩	2997	6.40	5.78
					+0.62*|23(1)⟩ -0.59*|16(1),15(1),1(1)⟩	3003	-11.29	2.30
					+1.00*|12(1),9(1),4(1)⟩	3004	-2.51	0.56
					+0.85*|11(1),10(1),4(1)⟩	3009	-9.70	6.90
					+0.85*|18(1),11(1),2(1)⟩	3009	-2.40	1.84
					+0.98*|17(1),5(1),4(1)⟩	3013	5.28	0.95
					+0.87*|11(1),7(2)⟩	3018	-17.47	9.67
					+1.00*|13(1),7(1),5(1)⟩	3018	-8.62	4.34
					+0.47*|11(1),7(2)⟩ -0.42*|20(1)⟩ + 0.39*|18(2)⟩	3019	-24.77	22.83
					+1.00*|18(1),10(1),3(1)⟩	3019	4.08	2.07
					+1.00*|13(1),8(1),4(1)⟩	3021	-0.27	2.06
					+1.00*|10(1),8(1),6(1)⟩	3022	1.10	1.93
					+0.76*|18(1),12(1),2(1)⟩ + 0.55*|11(2),4(1)⟩	3029	-1.16	1.06
					+0.97*|15(1),5(2)⟩	3030	-3.19	3.09
					+1.00*|12(1),10(1),4(1)⟩	3034	-8.59	5.43
					+0.88*|18(1),5(1),4(1)⟩	3035	-8.00	10.53
					+1.00*|14(1),13(1),3(1)⟩	3035	-3.80	1.57
					+1.00*|15(1),13(1),2(1)⟩	3037	-7.23	5.55
**III-3 3040-3290**					+1.00*|9(1),8(1),7(1)⟩	3047	-3.24	0.39
					+0.98*|17(1),16(1),1(1)⟩	3049	4.14	0.56
	**24**	3185	31.07	20.19	+0.99*|24(1)⟩	3054	52.72	23.03
					+1.00*|11(1),9(1),6(1)⟩	3124	2.26	0.50

[a]*The anharmonic value has been shifted by -18* cm^{-1}.

Table S4. Fundamental harmonic and corresponding anharmonic states and a short description of their vibrations.

Mode	Anharmonic analogue	Mode descriptions
1	+1.00*|1(1)⟩	*CH_3 hindered rotation*
2	+0.91*|2(1)⟩+0.42*|3(1)⟩	*Bend (C1-C4-C6 + O10-C4-C6)*
3	+0.91*|3(1)⟩-0.42*|2(1)⟩	*Bend (O10-C4-C6 + C1-C4-C6)*
4	+1.00*|4(1)⟩	*Ring deforming (C1-C4-O10))*
5	+1.00*|5(1)⟩	*Ring deforming (C1-C4-O10)*
6	+1.00*|6(1)⟩	*Wag (C1-H_2, C4-H, C6-H_3)*
7	+0.99*|7(1)⟩	*Str (C4-C6) +rock (CH_3)*
8	+1.00*|8(1)⟩	*Wag (C1-H_2, C4-H, C6-H_3)*
9	+0.99*|9(1)⟩	*Wag (C1-H_2, C4-H, C6-H_3)*
10	+0.99*|10(1)⟩	*Wag (CH_2)*
11	+0.98*|11(1)⟩	*Wag (C1-H2, C4-H5, C4-CH_3)*
12	+0.99*|12(1)⟩	*Wag (C1-H2H3, C4-H5, C4-CH_3)*
13	+1.00*|13(1)⟩	*Wag(C4-H5)*
14	+0.99*|14(1)⟩	*Sym. bend (CH_3)*
15	+0.97*|15(1)⟩	*Scissor (C2-H2H3, C6-H8H9), Bend(C4-H5)*
16	+0.76*|6(1),2(1),1(1)⟩-0.63*|16(1)⟩<br>+0.72*|16(1)⟩+0.63*|6(1),2(1),1(1)⟩	*Scissor (C1-H2H3, C6-H7H8)*
17	+0.91*|17(1)⟩	*Scissor (C1-H2H3, C6-H8H9)*
18	+0.75*|4(2)⟩+0.60*|18(1)⟩<br>+0.73*|18(1)⟩-0.64*|4(2)⟩	*Scissor (C1-H2H3)*
19	+0.68*|17(1),4(2)⟩ +0.47*|19(1)⟩ -0.44*|17(2)⟩<br>+0.64*|17(1),4(2)⟩-0.35*|19(1)⟩ +0.54*|16(1),9(1),3(1)⟩<br>+0.81*|16(1),9(1),3(1)⟩ +0.36*|19(1)⟩	*Sym. str. CH_3*
20	+0.88*|16(1),4(2)⟩<br>+0.52*|20(1)⟩ +0.48*|18(1),17(1)⟩<br>-0.47*|16(1),4(2)⟩ +0.45*|18(2)⟩	*Sym. str. CH_2*
21	+0.90*|21(1)⟩ -0.35*|17(1),9(1),3(1)⟩	*Asym. Str. (C6-H8+C6-H9)*
22	+0.72*|22(1)⟩ -0.45*|23(1)⟩	*Asym. Str (C6-H8+C6-H9), str (C4-H5)*
23	+0.75*|16(1),15(1),1(1)⟩+0.36*|22(1)⟩<br>+0.62*|23(1)⟩ -0.59*|16(1),15(1),1(1)⟩	*Asym. Str (C6-H7, C6-H8+C6-H9), str (C4-H5)*
24	+0.99*|24(1)⟩	*Asym. Str (C1-H2+C1-H3)*

Figure S5. wavenumbers (cm^{-1}). The units of ROA and Raman activities are $10^{-5}Å^6$ and 10^{-1}, respectively.

**Experimental range**	**Anharmonic**				
	ν	States	E.	I_{ROA}	I_{Raman}
**II-1 1700-1840**	119	+1.00*|16(1),1(2)⟩	1755	11.14	1.92
	121	+1.00*|8(1),4(1)⟩	1768	-8.89	0.91
	123	+1.00*|17(1),1(2)⟩	1777	-4.52	4.80
	124	+1.00*|6(2)⟩	1778	-23.13	2.74
	128	+1.00*|15(1),2(1)⟩	1788	-9.12	0.77
	132	+1.00*|16(1),2(1)⟩	1818	7.69	2.46
**II-2 1840-1990**	138	+1.00*|17(1),2(1)⟩	1845	-11.07	1.47
	148	+1.00*|17(1),3(1)⟩	1882	1.61	3.29
	150	+1.00*|7(2)⟩	1889	17.22	4.74
	156	+1.00*|8(1),6(1)⟩	1911	17.80	1.23
	159	+1.00*|12(1),4(1)⟩	1923	7.76	2.08
	170	+1.00*|10(1),5(1)⟩	1957	16.20	2.55
	173	+1.00*|8(1),7(1)⟩	1967	-7.07	0.61
**II-3 1990-2140**	180	+1.00*|9(1),6(1)⟩	1992	-7.29	0.64
	187	+1.00*|13(1),4(1)⟩	2019	7.22	1.08
	190	+1.00*|10(1),6(1)⟩	2022	15.33	1.58
	213	+1.00*|11(1),7(1)⟩	2097	-18.90	1.83
	217	+1.00*|12(1),7(1)⟩	2123	8.67	1.54
**II-4 2070-2298**	226	+1.00*|10(1),8(1)⟩	2153	-9.37	1.80
	229	+1.00*|13(1),6(1)⟩	2160	14.81	1.11
	245	+1.00*|9(2)⟩	2205	-12.37	2.07
	248	+1.00*|17(1),4(1)⟩	2210	-13.33	3.32
	251	+1.00*|13(1),7(1)⟩	2218	-9.32	1.58
	256	+1.00*|15(1),5(1)⟩	2231	14.72	3.06
	258	+1.00*|18(1),4(1)⟩	2237	24.79	2.06
	267	+1.00*|11(1),9(1)⟩	2256	18.08	0.90
	269	+1.00*|14(1),6(1)⟩	2257	-5.67	3.81
	270	+1.00*|10(2)⟩	2262	-47.07	8.00
	280	+1.00*|11(1),10(1)⟩	2285	11.13	1.71
	288	+1.00*|15(1),6(1)⟩	2298	25.05	7.01
**II-5 2298-2440**	294	+1.00*|11(2)⟩	2304	-11.95	1.95
	295	+1.00*|12(1),10(1)⟩	2309	-11.14	1.29
	296	+1.00*|18(1),5(1)⟩	2310	-13.93	2.40
	298	+1.00*|14(1),7(1)⟩	2313	12.63	4.08

	299	+1.00*|11(1),4(1),3(1)⟩	2314	-7.72	1.04
	304	+1.00*|12(1),11(1)⟩	2329	-9.39	6.72
	305	+1.00*|16(1),6(1)⟩	2329	-39.22	5.17
	312	+1.00*|12(2)⟩	2351	-8.46	3.13
	313	+1.00*|17(1),6(1)⟩	2351	29.35	1.27
	314	+1.00*|15(1),7(1)⟩	2354	-16.01	5.87
	320	+1.00*|9(1),6(1),2(1)⟩	2371	-10.61	0.51
	324	+1.00*|18(1),6(1)⟩	2378	-8.21	5.72
	328	+1.00*|14(1),8(1)⟩	2387	6.76	5.96
	336	+1.00*|13(1),10(1)⟩	2405	8.64	3.22
	339	+1.00*|17(1),7(1)⟩	2409	-1.11	5.58
	343	+1.00*|11(1),6(1),2(1)⟩	2422	-10.88	0.43
	347	+1.00*|15(1),8(1)⟩	2430	0.81	4.81
	354	+1.00*|18(1),7(1)⟩	2438	-6.29	3.49
	356	+1.00*|13(1),12(1)⟩	2448	16.29	3.10
**II-6 2440-2570**	365	+1.00*|16(1),8(1)⟩	2462	-16.18	4.39
	370	+1.00*|14(1),9(1)⟩	2471	-2.26	3.31
	376	+1.00*|17(1),8(1)⟩	2485	3.37	3.39
	384	+1.00*|14(1),10(1)⟩	2503	-14.66	0.40
	387	+1.00*|15(1),9(1)⟩	2512	-11.56	9.66
	392	+1.00*|14(1),11(1)⟩	2520	-14.69	5.71
	403	+1.00*|13(2)⟩	2542	4.40	7.09
	404	+1.00*|16(1),9(1)⟩	2542	46.80	2.85
	405	+1.00*|15(1),10(1)⟩	2544	-3.44	3.68
	406	+1.00*|14(1),12(1)⟩	2544	27.02	2.89
	412	+1.00*|15(1),11(1)⟩	2562	4.76	5.13
**II-7 2570-2700**	415	+1.00*|17(1),9(1)⟩	2566	-20.92	4.35
	422	+1.00*|17(1),7(1),1(1)⟩	2579	-11.14	2.35
	424	+1.00*|9(2),2(1)⟩	2583	16.68	0.86
	425	+1.00*|15(1),12(1)⟩	2585	-18.44	6.05
	430	+1.00*|18(1),9(1)⟩	2591	-15.19	20.55
	432	+1.00*|16(1),11(1)⟩	2592	-4.99	5.22
	436	+1.00*|17(1),10(1)⟩	2598	9.44	0.87
	445	+1.00*|17(1),11(1)⟩	2616	37.12	4.41
	446	+1.00*|16(1),12(1)⟩	2617	-11.44	1.95
	450	+1.00*|18(1),10(1)⟩	2623	-18.49	10.04
	452	+1.00*|17(1),4(1),3(1)⟩	2628	9.38	0.99
	456	+1.00*|11(1),9(1),2(1)⟩	2637	17.84	1.93

<table>
<tr><td rowspan="3"></td><td>459</td><td>+1.00*|17(1),12(1)⟩</td><td>2640</td><td>-32.37</td><td>2.82</td></tr>
<tr><td>472</td><td>+1.00*|18(1),12(1)⟩</td><td>2665</td><td>1.45</td><td>19.06</td></tr>
<tr><td>484</td><td>+1.00*|15(1),13(1)⟩</td><td>2678</td><td>0.84</td><td>17.69</td></tr>
<tr><td rowspan="23">II-8
2700-2900</td><td>503</td><td>+1.00*|12(1),11(1),2(1)⟩</td><td>2712</td><td>12.09</td><td>0.93</td></tr>
<tr><td>504</td><td>+1.00*|16(1),13(1)⟩</td><td>2715</td><td>-8.31</td><td>0.81</td></tr>
<tr><td>511</td><td>+1.00*|14(2)⟩</td><td>2723</td><td>-28.68</td><td>151.90</td></tr>
<tr><td>522</td><td>+1.00*|17(1),13(1)⟩</td><td>2736</td><td>-7.60</td><td>3.53</td></tr>
<tr><td>534</td><td>+1.00*|18(1),13(1)⟩</td><td>2763</td><td>17.36</td><td>10.20</td></tr>
<tr><td>536</td><td>+1.00*|14(1),8(1),2(1)⟩</td><td>2764</td><td>-1.27</td><td>4.11</td></tr>
<tr><td>542</td><td>+1.00*|15(1),14(1)⟩</td><td>2771</td><td>-17.40</td><td>32.11</td></tr>
<tr><td>548</td><td>+1.00*|11(1),6(1),4(1)⟩</td><td>2785</td><td>8.12</td><td>0.31</td></tr>
<tr><td>560</td><td>+1.00*|16(1),14(1)⟩</td><td>2804</td><td>17.79</td><td>10.82</td></tr>
<tr><td>565</td><td>+0.98*|15(2)⟩</td><td>2810</td><td>-178.60</td><td>187.10</td></tr>
<tr><td>576</td><td>+0.99*|17(1),14(1)⟩</td><td>2829</td><td>-6.84</td><td>28.28</td></tr>
<tr><td>582</td><td>+1.00*|15(1),8(1),3(1)⟩</td><td>2841</td><td>-2.91</td><td>11.08</td></tr>
<tr><td>585</td><td>+1.00*|16(1),15(1)⟩</td><td>2850</td><td>33.24</td><td>19.00</td></tr>
<tr><td>590</td><td>+0.81*|16(2)⟩+0.45*|19(1)⟩</td><td>2860</td><td>23.47</td><td>717.20</td></tr>
<tr><td>592</td><td>+1.00*|18(1),14(1)⟩</td><td>2861</td><td>9.18</td><td>1.55</td></tr>
<tr><td>597</td><td>+1.00*|17(1),15(1)⟩</td><td>2866</td><td>-130.90</td><td>99.87</td></tr>
<tr><td>598</td><td>+1.00*|9(1),8(1),4(1)⟩</td><td>2867</td><td>11.64</td><td>0.79</td></tr>
<tr><td>600</td><td>+1.00*|16(1),8(1),3(1)⟩</td><td>2874</td><td>6.10</td><td>6.66</td></tr>
<tr><td>606</td><td>+1.00*|15(1),9(1),2(1)⟩</td><td>2889</td><td>-6.22</td><td>10.52</td></tr>
<tr><td>608</td><td>+1.00*|14(2),1(1)⟩</td><td>2894</td><td>-0.67</td><td>14.07</td></tr>
<tr><td>609</td><td>+1.00*|18(1),15(1)⟩</td><td>2895</td><td>186.50</td><td>106.10</td></tr>
<tr><td>610</td><td>+1.00*|10(1),8(1),4(1)⟩</td><td>2897</td><td>8.50</td><td>0.88</td></tr>
<tr><td>611</td><td>+1.00*|15(1),4(2)⟩</td><td>2897</td><td>-4.12</td><td>3.77</td></tr>
</table>

Table S6. Transitions within 3290-4300 cm^{-1}. Symbols are the same in Table S4.

Experimental range	Anharmonic				
	v	States	E.	I_{ROA}	I_{Raman}
IV-1 3290-3508	884	+1.00*|21(1),1(2)⟩	3306	12.71	4.56
	928	+1.00*|21(1),2(1)⟩	3361	2.29	9.59
	937	+1.00*|20(1),2(1)⟩	3370	-8.50	2.81
	942	+1.00*|22(1),2(1)⟩	3378	27.44	2.70
	953	+1.00*|23(1),2(1)⟩	3395	-45.30	3.77
	959	+1.00*|21(1),3(1)⟩	3398	20.42	4.41
	966	+1.00*|20(1),3(1)⟩	3406	-12.43	4.52
	973	+1.00*|22(1),3(1)⟩	3416	-37.85	3.22
	983	+1.00*|23(1),3(1)⟩	3432	15.86	9.22
	993	+1.00*|24(1),2(1)⟩	3450	46.64	10.90
	1022	+1.00*|24(1),3(1)⟩	3487	-7.49	5.38
IV-2 3508-3630	1071	+1.00*|23(1),2(1),1(1)⟩	3564	-7.45	8.59
	1072	+1.00*|21(1),3(1),1(1)⟩	3568	-0.45	5.47
IV-3 3630-3800	1173	+1.00*|21(1),4(1)⟩	3731	-10.60	2.47
	1189	+1.00*|22(1),4(1)⟩	3748	-14.44	1.29
	1202	+1.00*|23(1),4(1)⟩	3764	2.01	4.42
	1230	+1.00*|21(1),5(1)⟩	3808	15.80	3.91
	1241	+1.00*|24(1),4(1)⟩	3819	-11.88	2.64
	1242	+1.00*|19(1),6(1)⟩	3819	6.10	3.34
IV-4 3800-3920	1271	+1.00*|21(1),6(1)⟩	3872	-2.19	7.33
	1275	+1.00*|20(1),6(1)⟩	3876	-6.34	5.56
	1282	+1.00*|22(1),6(1)⟩	3889	29.38	1.17
	1283	+1.00*|24(1),5(1)⟩	3894	2.96	3.74
	1289	+1.00*|23(1),6(1)⟩	3904	-5.91	9.31
IV-5 3920-4060	1319	+1.00*|24(1),6(1)⟩	3957	-12.93	12.93
	1321	+1.00*|23(1),7(1)⟩	3963	-8.85	5.31
	1342	+1.00*|21(1),8(1)⟩	4003	-17.64	5.45
	1346	+1.00*|20(1),8(1)⟩	4010	-8.30	3.89
	1351	+1.00*|22(1),8(1)⟩	4019	-8.90	2.09
	1362	+1.00*|23(1),8(1)⟩	4035	33.96	5.34
IV-6 4060-4185	1390	+1.00*|20(1),9(1)⟩	4091	-2.63	3.04
	1391	+1.00*|24(1),8(1)⟩	4091	-3.75	4.30
	1408	+1.00*|23(1),9(1)⟩	4120	-10.66	0.33
	1410	+1.00*|20(1),10(1)⟩	4124	3.54	10.89
	1418	+1.00*|22(1),10(1)⟩	4135	-10.82	3.44
	1422	+1.00*|20(1),11(1)⟩	4141	-2.59	4.94

	1425	+1.00*|22(1),11(1)⟩	4151	-54.11	4.09
	1431	+1.00*|21(1),12(1)⟩	4160	18.46	0.49
	1433	+1.00*|22(1),4(1),3(1)⟩	4162	-11.71	1.18
	1435	+1.00*|23(1),11(1)⟩	4167	27.28	2.44
	1439	+1.00*|24(1),9(1)⟩	4173	7.17	9.65
	1440	+1.00*|22(1),12(1)⟩	4173	4.13	8.31
	1446	+1.00*|23(1),12(1)⟩	4190	-40.18	2.93
	1453	+1.00*|24(1),10(1)⟩	4199	-5.30	8.85
IV-7 4185-4300	1463	+1.00*|24(1),11(1)⟩	4221	6.70	3.44
	1472	+1.00*|24(1),12(1)⟩	4245	27.39	3.55
	1489	+1.00*|22(1),13(1)⟩	4269	21.31	7.19
	1497	+1.00*|23(1),13(1)⟩	4286	13.21	2.98

Figure S1 Anharmonic spectra calculated by including (Default) or not (NoLAM) the contribution from the methyl torsional mode in the anharmonic correction.

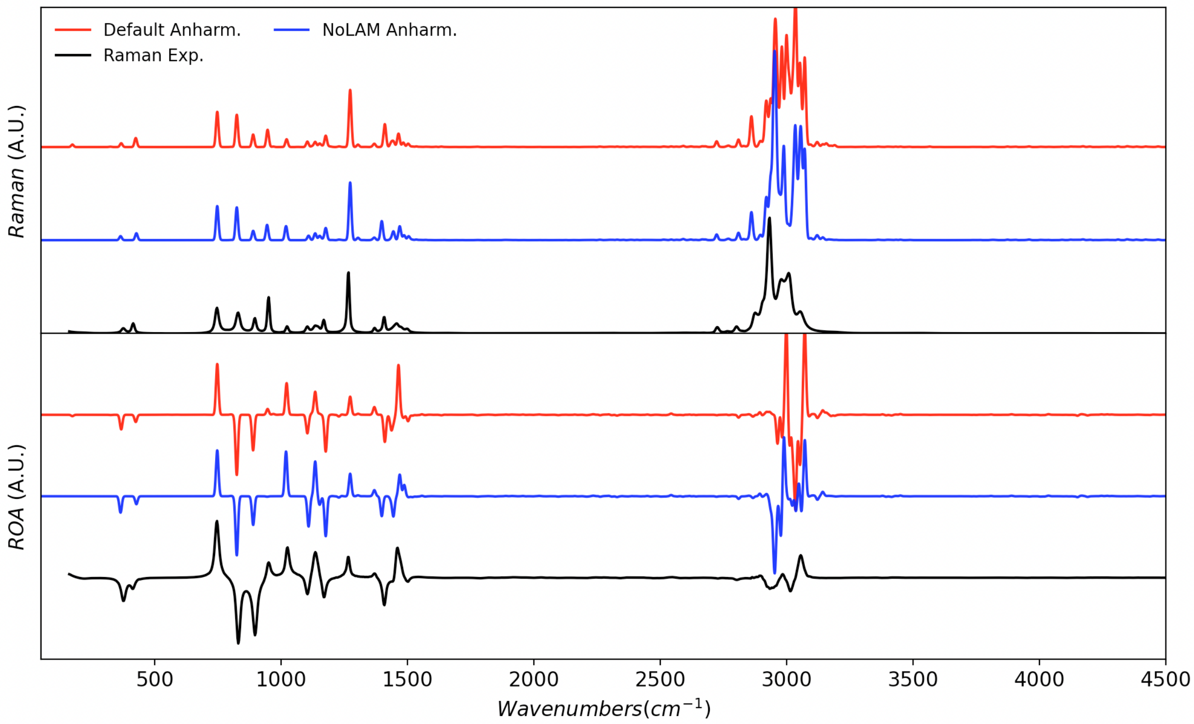

Figure S2. Mean (MAE) and maximum (|MAX|) absolute errors for the electronic structure calculation methods. ω (clear blue/blue) corresponds to the harmonic approximation, ν (pink/red) to the anharmonic level. Gas phase experimental energies are taken from Ref.[20-22] B2PD/JNTZ// B3PD/JNTZ, RDSD/JNTZ// B3PD/JNTZ and CCSD/ATZ// B3P/JNTZ are hybrid schemes based on B3PD/JNTZ for the anharmonic part. The left panel gives the error on the whole energy range, and the right panel only for fundamentals below 1600 cm^{-1}.

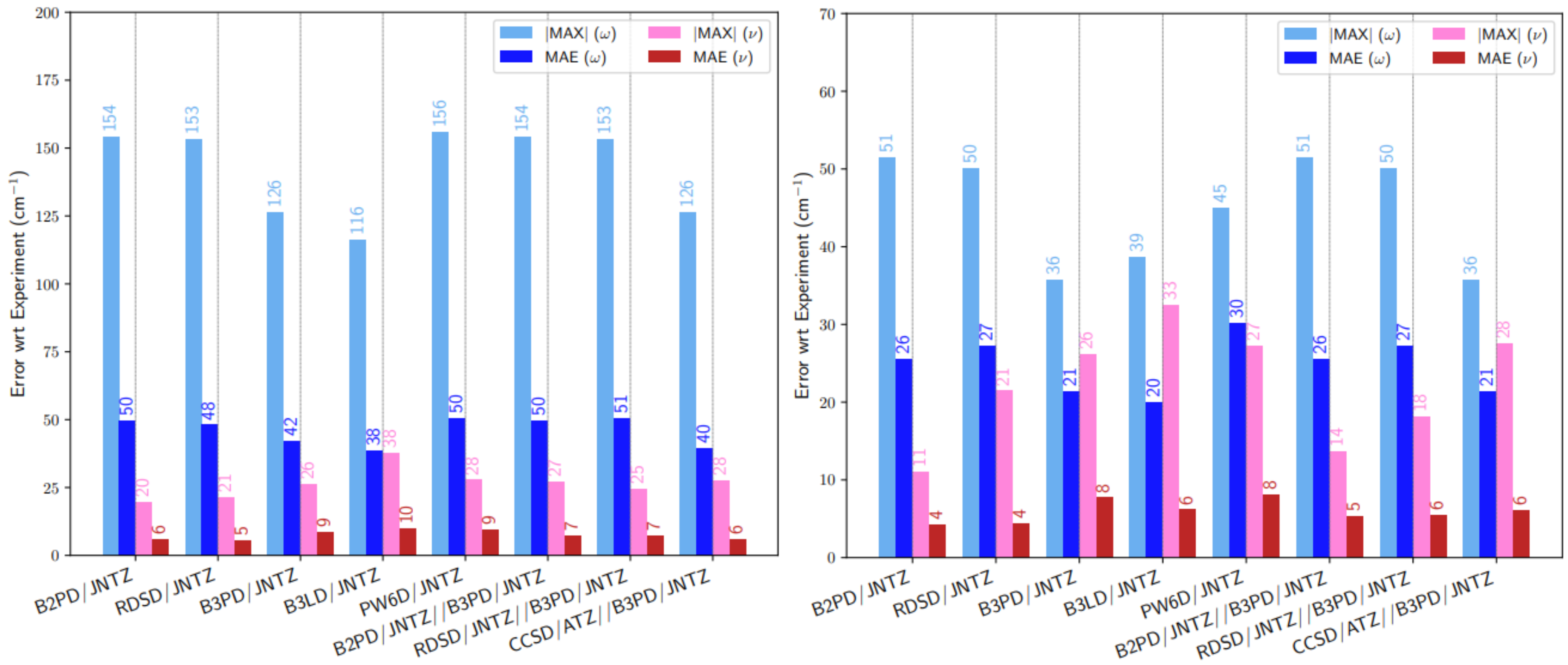

Figure S3 Solvent effects. Spectra were simulated with 2-hexaneone (ε=11.66), dichloromethane (ε=8.93), pentanal (ε=10.00), and tetrahydrofuran (ε=7.46).

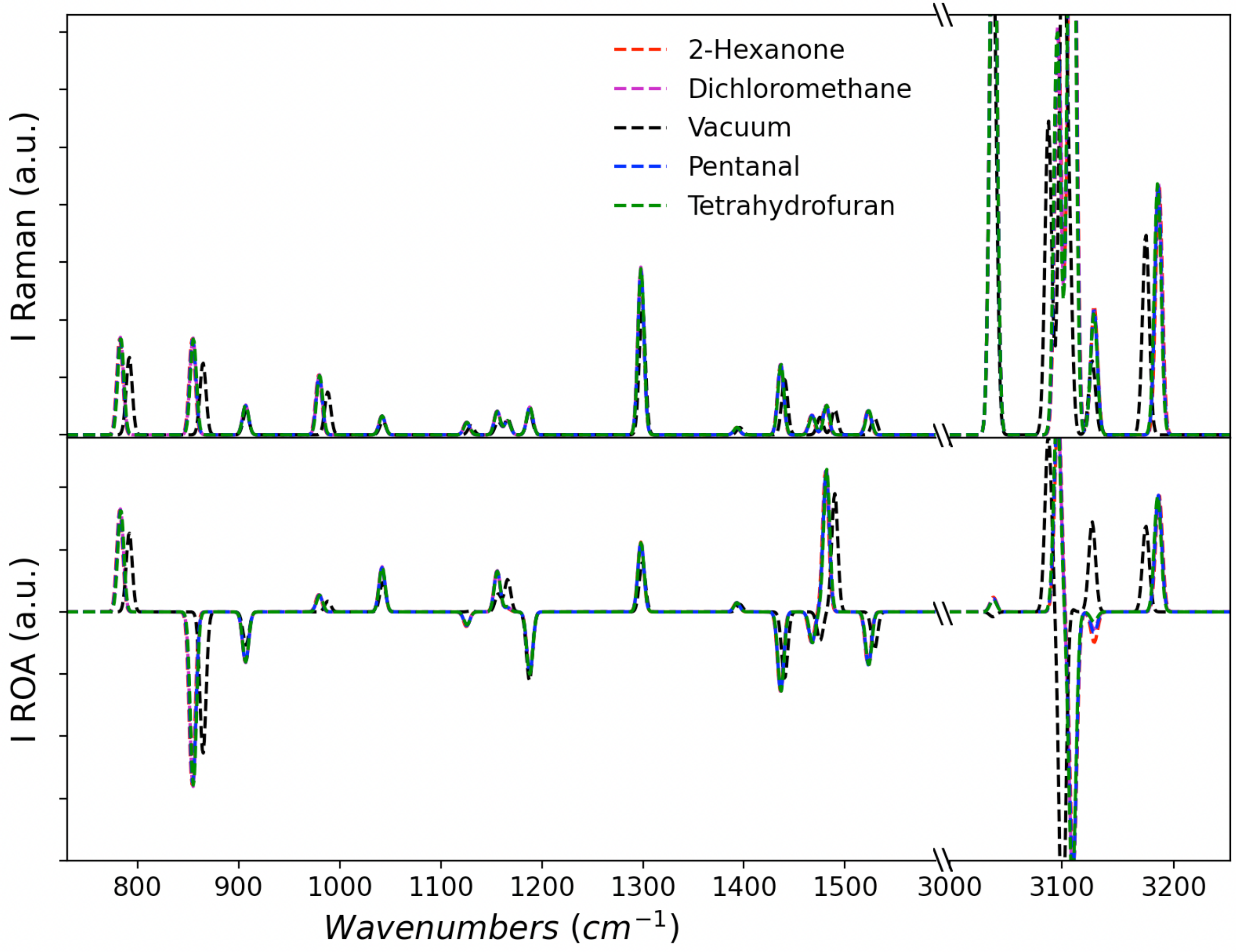

Figure S4. Spectra simulated including property-related (blue) and mechanical (green) anharmonicities. The upper panel includes the whole range, the lower panel is a zoom of a part without fundamental transition ("zone II").

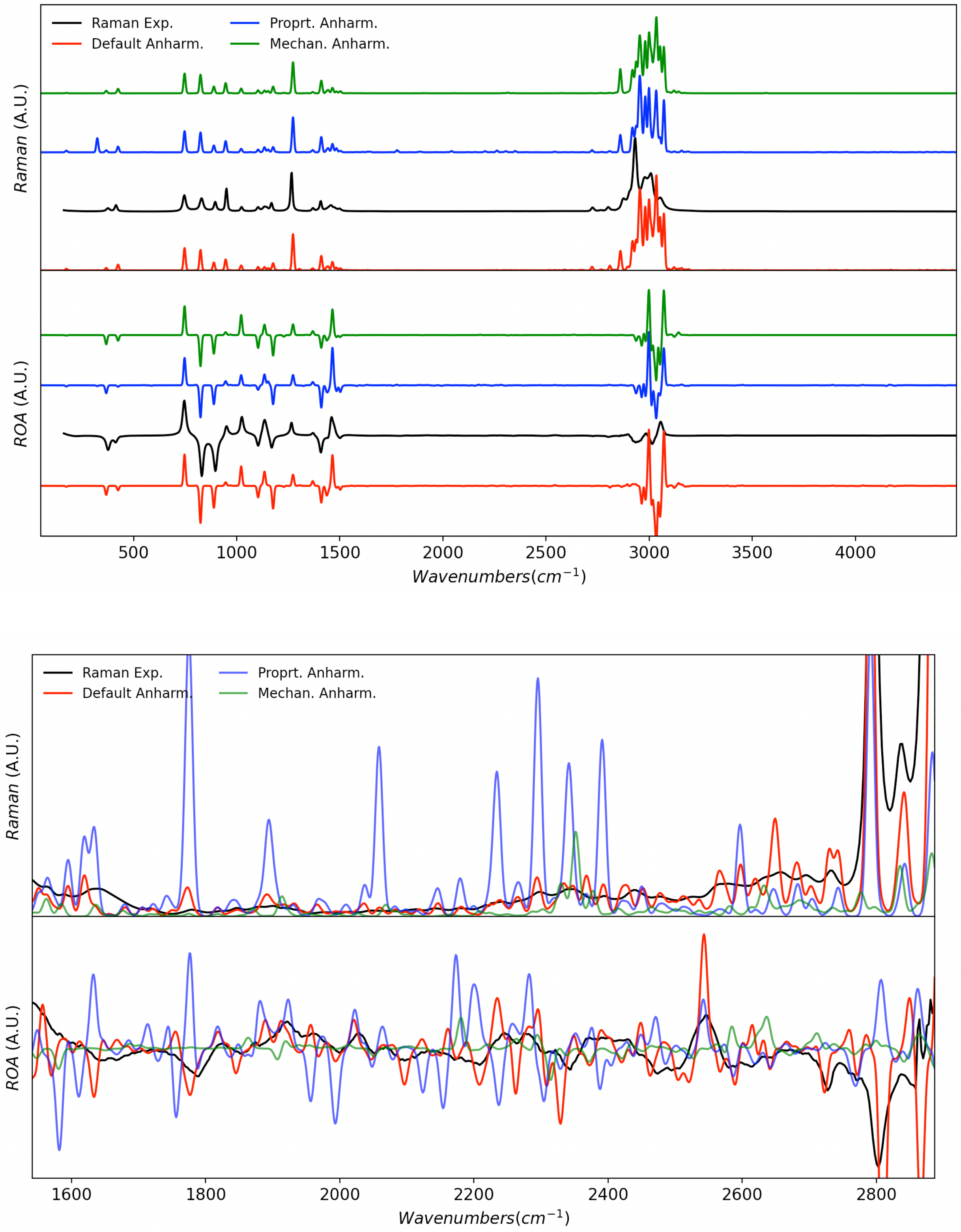

Figure S5. Influence of the Coriolis couplings on the total anharmonic contributions. The upper panel include the whole spectra, the lower panel is a zoom of a part without fundamental transition ("zone II").

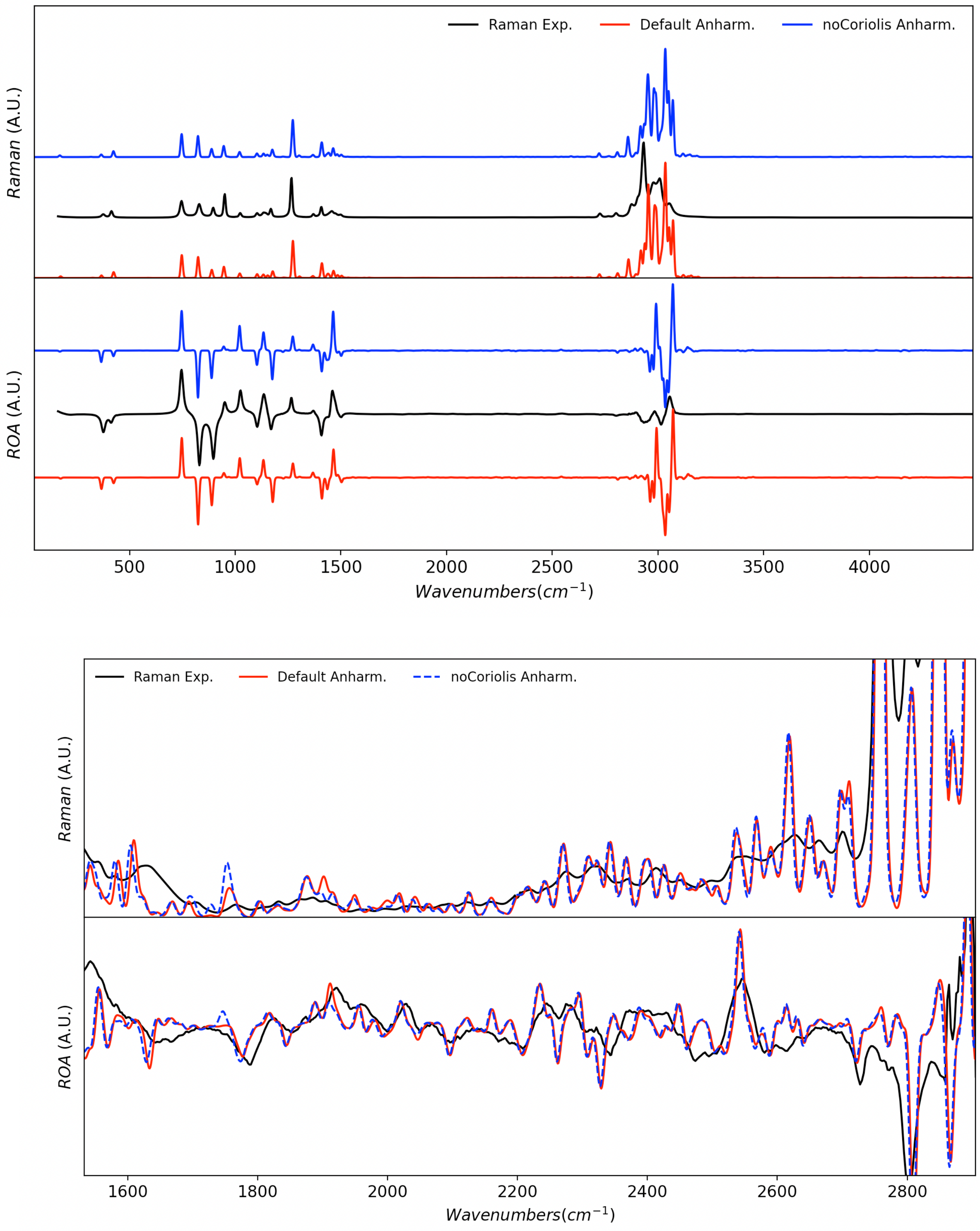

Figure S6. Simulated and experimental spectra of R-methyloxirane in the 150-4300 cm^{-1} region. For better visualization, the harmonic and anharmonic spectra of III and IV have been shifted along the y-axis. The theoretical intensities were normalized based on the Raman experiment, with the ROA/Raman ratio conserved.

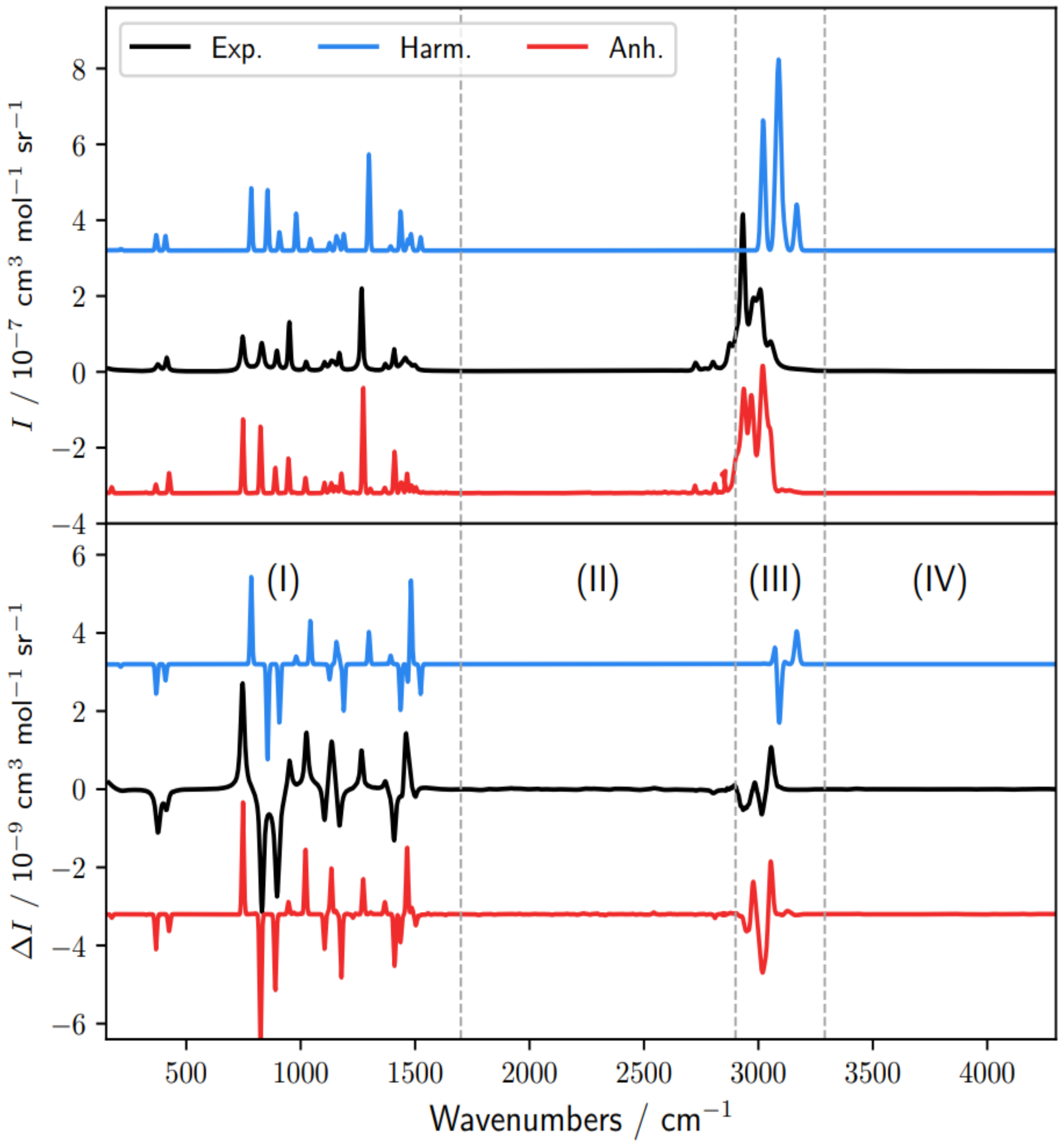

Figure S7. Simulated and experimental spectra of R-methyloxirane within 150-1700 cm^{-1}.

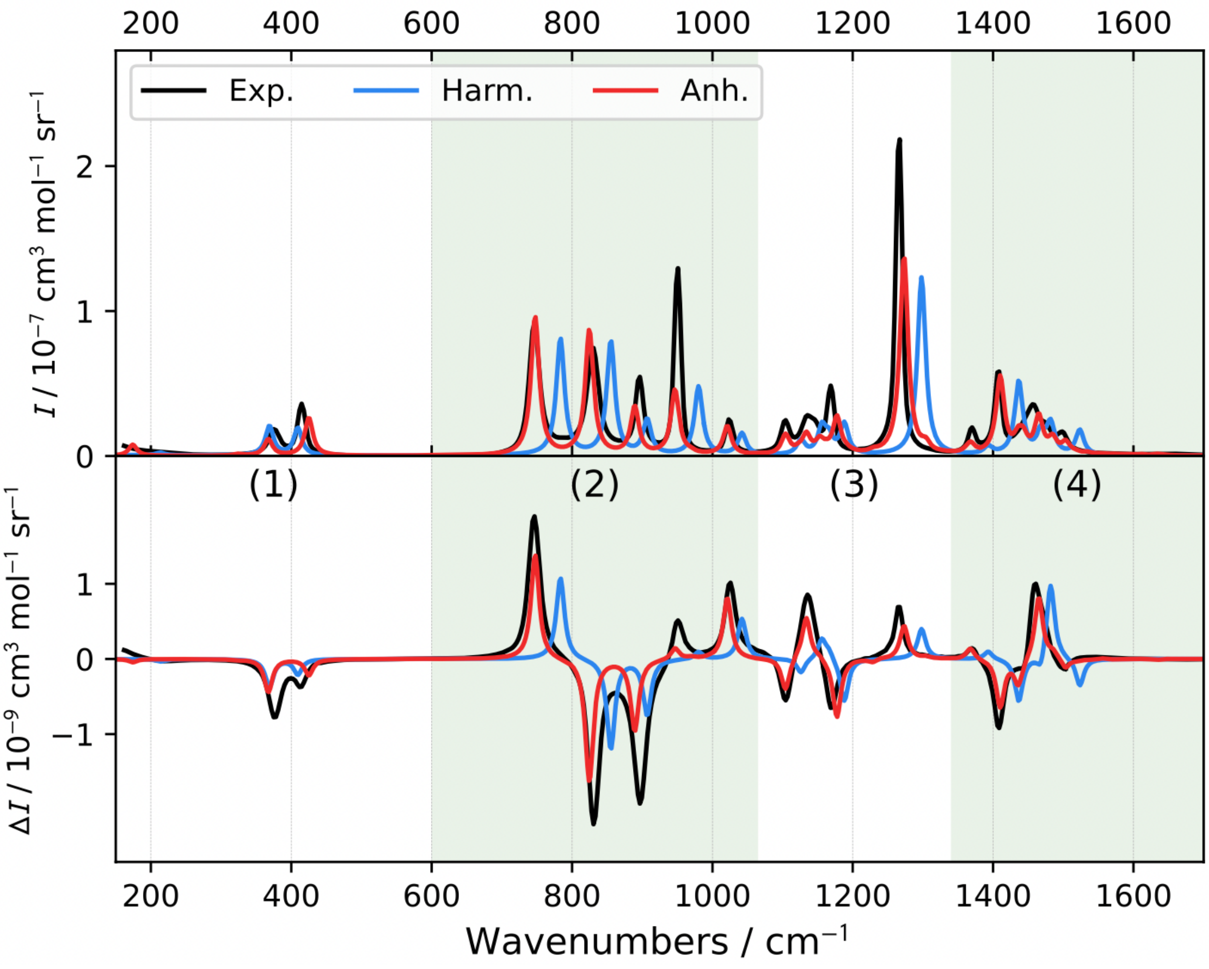

Figure S8. Anharmonic and harmonic frequency errors for the modes 1-18. the standard deviation is 25 cm^{-1} and 6 cm^{-1} for the harmonic and anharmonic calculations, respectively.

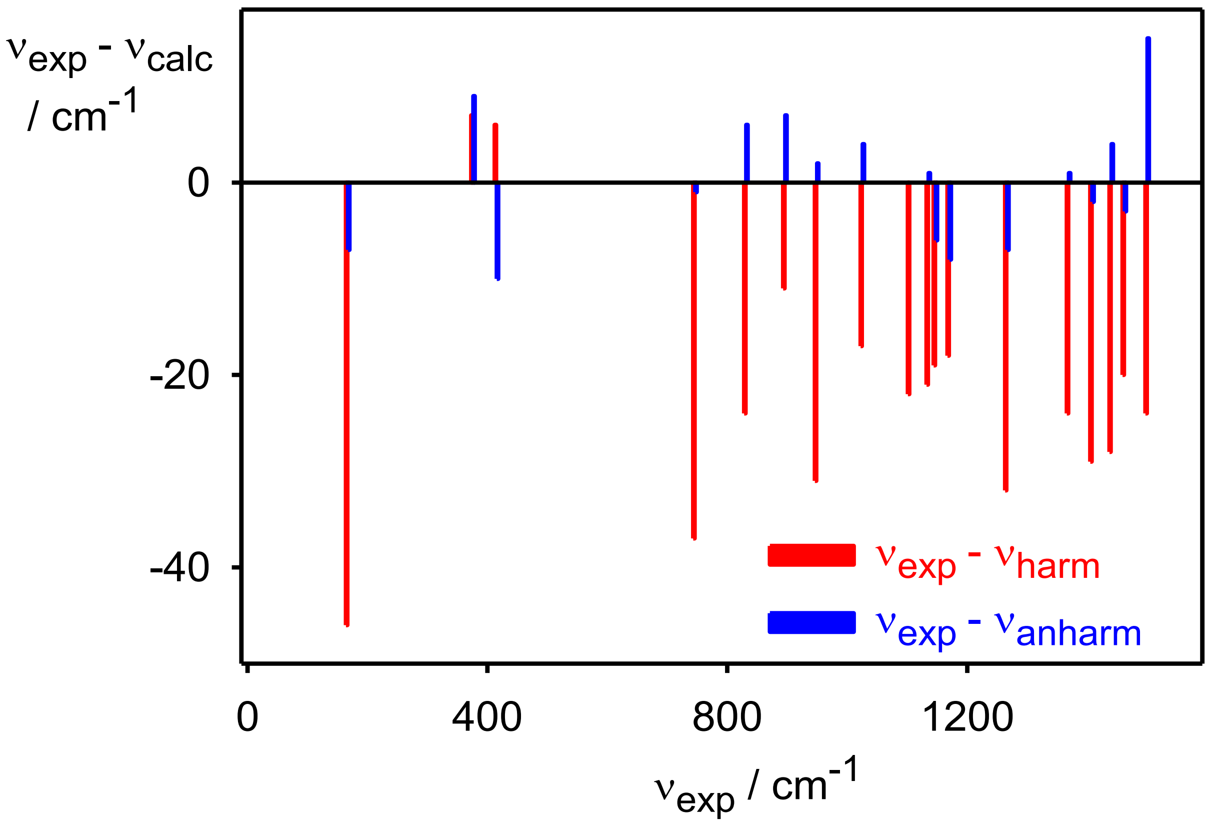

Figure S9. Simulated and experimental spectra of R-methyloxirane within 2700-3290 cm^{-1}.

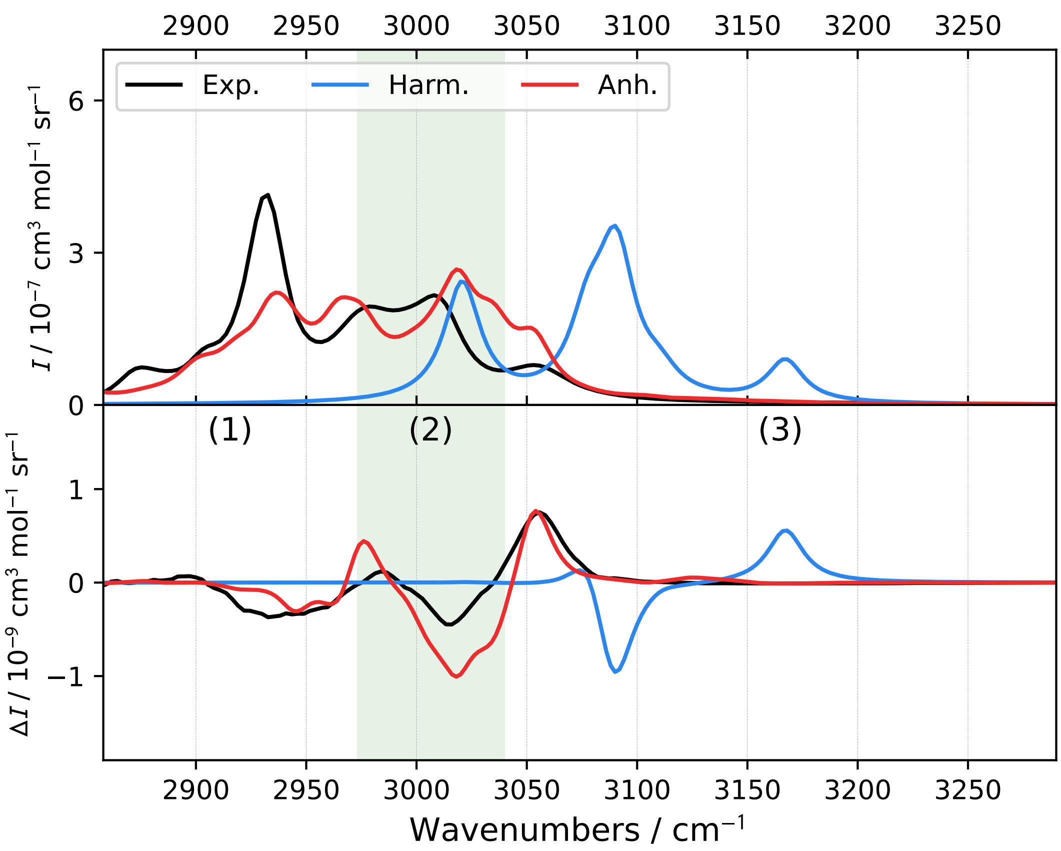

Figure S10. The **J** matrix (upper panel) and shift vector (**K**, lower panel) representing the changes between the normal modes and the shift between the equilibrium structures computed at the B3PW91-D3(BJ)/jun-cc-pVTZ and rev-DSD-PBEP86-D3(BJ)/jun-cc-pVTZ calculations, respectively. The elements of **J** have been squared and a value of gray assigned to each one, from white (squared value equal to 0) to black (1). The shift vector is in atomic units, and displayed as absolute value. The color represents the sign (blue: positive, red: negative).

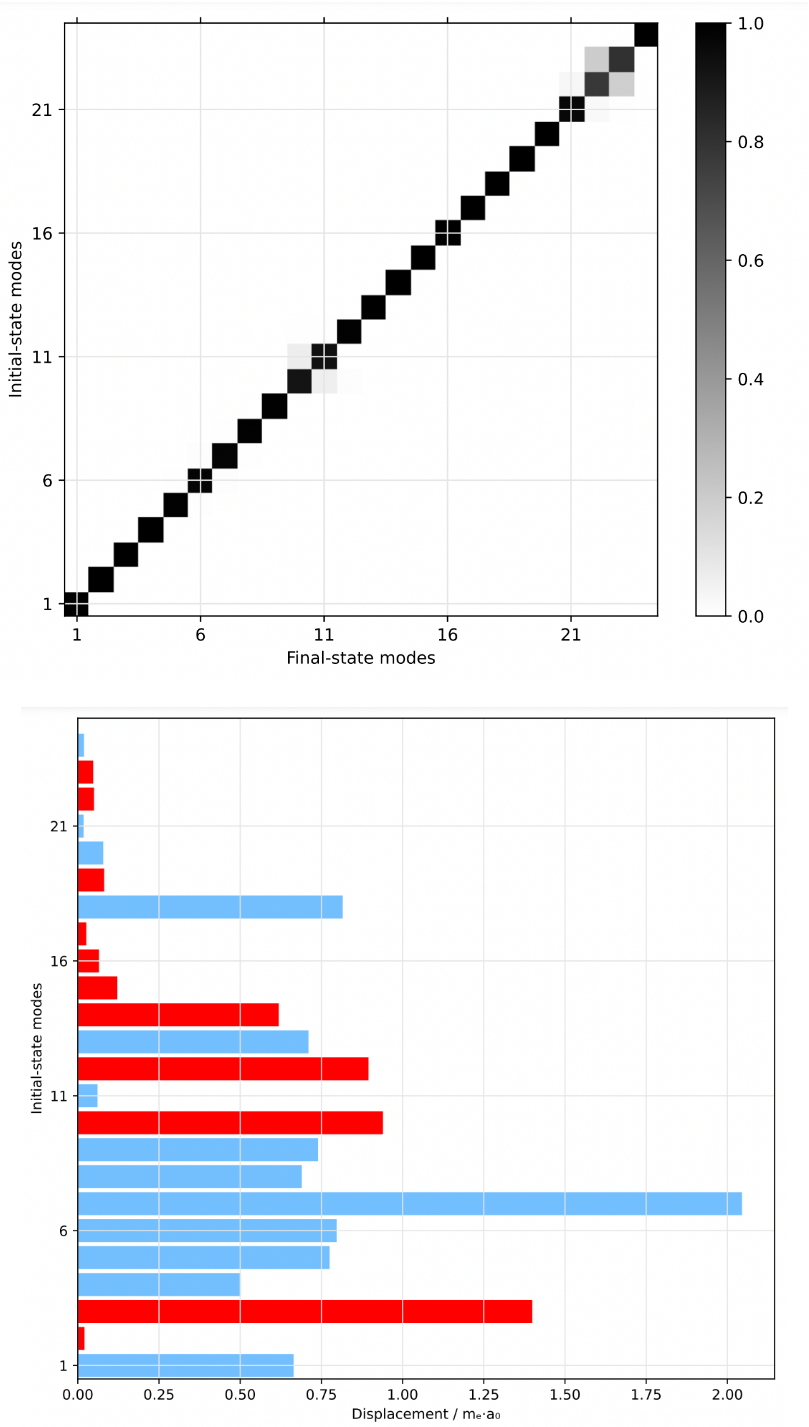

Figure S11. Influence of the threshold used to identify 1-1 DDRs on the band-shape.

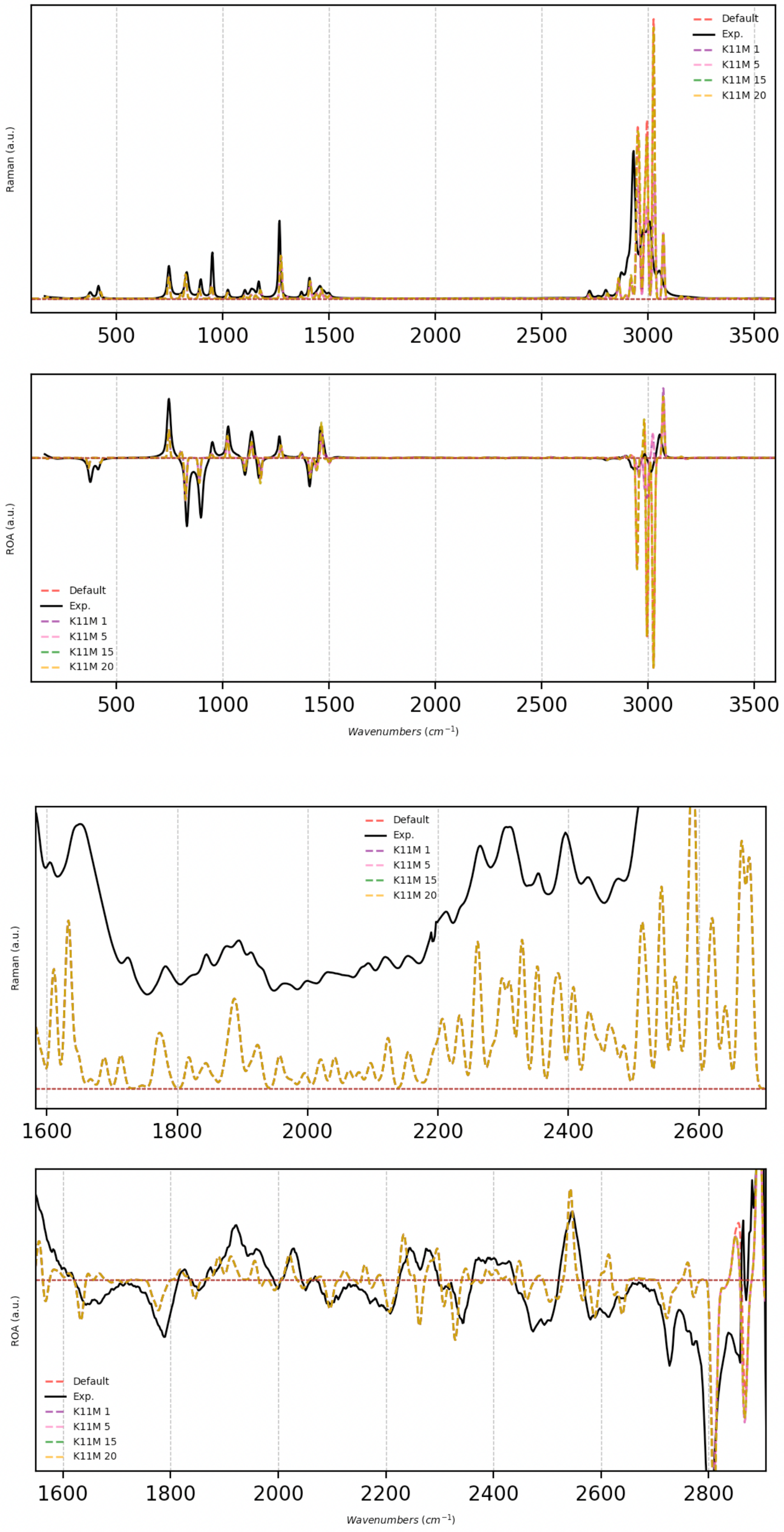

References

(1) Michal, P.; Čelechovský, R.; Dudka, M.; Kapitán, J.; Vůjtek, M.; Berešová, M.; Sebestik, J.; Thangavel, K.; Bouř, P. Vibrational optical activity of intermolecular, overtone, and combination bands: 2-chloropropionitrile and α-pinene. *J. Phys. Chem. B* **2019**, *123* (9), 2147-2156.

(2) Hug, W.; Hangartner, G. A novel high‐throughput Raman spectrometer for polarization difference measurements. *J. Raman Spectrosc.* **1999**, *30* (9), 841-852.

(3) Hug, W. Virtual enantiomers as the solution of optical activity's deterministic offset problem. *Appl. Spectrosc.* **2003**, *57* (1), 1-13.

(4) Frisch, M.; Trucks, G.; Schlegel, H.; Scuseria, G.; Robb, M.; Cheeseman, J.; Scalmani, G.; Barone, V.; Mennucci, B.; Petersson, G.; et al. Gaussian Development Version, Revision J13. *Wallingford Ct* **2022**.

(5) Barone, V. Anharmonic vibrational properties by a fully automated second-order perturbative approach. *J. Chem. Phys.* **2005**, *122* (1), 014108.

(6) Duschinsky, F. On the interpretation of electronic spectra of polyatomic molecules. *Acta Physicochim. U.R.S.S.* **1937**, *7*, 551.

(7) Yang, Q.; Mendolicchio, M.; Barone, V.; Bloino, J. Accuracy and Reliability in the Simulation of Vibrational Spectra: A Comprehensive Benchmark of Energies and Intensities issuing from Generalized Vibrational Perturbation Theory to Second Order (GVPT2). *Front. Astron. Space Sci.* **2021**, *8*, 77.

(8) Bloino, J.; Baiardi, A.; Biczysko, M. Aiming at an accurate prediction of vibrational and electronic spectra for medium‐to‐large molecules: an overview. *Int. J. Quantum Chem.* **2016**, *116* (21), 1543-1574.

(9) Bloino, J.; Biczysko, M.; Barone, V. General perturbative approach for spectroscopy, thermodynamics, and kinetics: Methodological background and benchmark studies. *J. Chem. Theory Comput.* **2012**, *8* (3), 1015-1036.

(10) Martin, J. M.; Taylor, P. R. Accurate ab initio quartic force field for trans-HNNH and treatment of resonance polyads. *Spectrochim. Acta A* **1997**, *53* (8), 1039-1050.

(11) Rosnik, A. M.; Polik, W. F. VPT2+K spectroscopic constants and matrix elements of the transformed vibrational Hamiltonian of a polyatomic molecule with resonances using Van Vleck perturbation theory. *Mol. Phys.* **2014**, *112* (2), 261-300.

(12) Grimme, S. Semiempirical hybrid density functional with perturbative second-order correlation. *J. Chem. Phys.* **2006**, *124* (3), 034108.

(13) Santra, G.; Sylvetsky, N.; Martin, J. M. Minimally empirical double-hybrid functionals trained against the GMTKN55 database: revDSD-PBEP86-D4, revDOD-PBE-D4, and DOD-SCAN-D4. *J. Phys. Chem. A* **2019**, *123* (24), 5129-5143.

(14) Goerigk, L.; Grimme, S. Double-hybrid density functionals. *Wiley Interdiscip. Rev. Comput. Mol. Sci.* **2014**, *4* (6), 576-600.

(15) Becke, A. Density-functional thermochemistry. III. The role of exact exchange *J. Chem. Phys.* **1993**, *98*, 5648.

(16) Zhao, Y.; Truhlar, D. G. Design of density functionals that are broadly accurate for thermochemistry, thermochemical kinetics, and nonbonded interactions. *J. Phys. Chem. A* **2005**, *109* (25), 5656-5667.

(17) Grimme, S.; Ehrlich, S.; Goerigk, L. Effect of the damping function in dispersion corrected density functional theory. *J. Comput. Chem.* **2011**, *32* (7), 1456-1465.

(18) Wiberg, K. B. Basis set effects on calculated geometries:6-311++G**vs.aug-cc-pVDZ. *J. Comput. Chem.* **2004**, *25* (11), 1342-1346.

(19) Papajak, E.; Zheng, J.; Xu, X.; Leverentz, H. R.; Truhlar, D. G. Perspectives on basis sets beautiful: Seasonal plantings of diffuse basis functions. *J. Chem. Theory Comput.* **2011**, *7* (10), 3027-3034.

(20) Merten, C.; Bloino, J.; Barone, V.; Xu, Y. Anharmonicity effects in the vibrational CD spectra of propylene oxide. *J. Phys. Chem. Lett.* **2013**, *4* (20), 3424-3428.

(21) Sunahori, F. X.; Su, Z.; Kang, C.; Xu, Y. Infrared diode laser spectroscopic investigation of four C–H stretching vibrational modes of propylene oxide. *Chem. Phys. Lett.* **2010**, *494* (1-3), 14-20.

(22) Polavarapu, P.; Hess Jr, B.; Schaad, L. Vibrational spectra of epoxypropane. *J. Chem. Phys.* **1985**, *82* (4), 1705-1710.
