# Supplementary material for: Anharmonic Vibrational Raman Optical Activity of Methyloxirane: Theory and Experiment Pushed to the Limits
Source: J Phys Chem Lett. 2022 Sep 20;13(38):8888–92. doi: 10.1021/acs.jpclett.2c02320 (PMC9531246; doi:10.1021/acs.jpclett.2c02320)
Supplement: Supplementary file 2 — jz2c02320_si_002.pdf [file jz2c02320_si_002.pdf]

jz-2022-02320x.R1

Name: Peer Review Information for "Anharmonic Vibrational Raman Optical Activity of Methyloxirane: Theory and Experiment Pushed to the Limits"

First Round of Reviewer Comments

Reviewer: 1

Comments to the Author

The manuscript indeed demonstrates how pushing experiment and theory into its limits allow obtaining unprecedented accuracy and very good agreement between both. The results and approaches presented in the paper are clearly not only state-of-the-art but rather a breakthrough in the chiroptical spectroscopies, opening new pathways for unbiased analysis many important biologically active molecules. For these reasons manuscript fully deserve to be published in the JPCL in the present form.

Manuscript is well written, provides all necessary details on computations and presents the results by very clear figures. I also appreciate that in addition to the main body of the text, which clearly present all most important aspects relevant for the broader audience, authors performed also detailed analysis of specific terms/effects affecting the final simulated spectra, which are very interesting for researchers working in the field.

I only noticed that the text Harm/Anharm is reversed in the legend to the figure S7 (colour code is OK).

Reviewer: 2

Comments to the Author

The manuscript by Yang et al. explores the current possibilities in structural characterization of small molecules (exemplified by propylene oxide) via a combination of Raman and ROA experiments and high-level anharmonic vibrational calculations. It provides an instructive demonstration of the currently available level of computational accuracy and, importantly, analyzes relative importance of different effects/contributions with regard to the final result. I believe that the findings of the present work will be of direct interest to the computational and spectroscopic communities and insightful to a broader range of physicochemists. Thus, the manuscript deserves to be published, but I would like the authors to address the following points:

1) The authors mention that the solvent effects have been taken into account via PCM. However, the use of the PCM normally reduces the achievable accuracy of gradients. I would like to see more discussion in that regard.

2) In the ESI, the authors correctly point out that different levels of theory can be reasonably combined only when the Duschinsky rotation and the shift vector are not large. It would be nice to have a table with characteristic parameters of the Duschinsky transformation between different levels of theory employed (and, perhaps, some related discussion).

3) The findings seem to demonstrate that anharmonic corrections are not of very high importance to the fundamentals (except for C-H stretching, naturally) where simple scaling might suffice. Much more interesting is the possibility to explore the combination/overtone fingerprints. While the figures reveal apparent qualitative agreement between the experiment and theory, one would wonder, how critically would the computational results depend on the variations in the anharmonicity parameters? How important is their accuracy?

Reviewer: 3

Comments to the Author

The authors report a combined theoretical and experimental study of Raman and Raman Optical Activity (ROA) spectra of methyloxirane in a much wider frequency range than normally conducted in these experiments due to experimental limitations. This is combined with an automated approach for the theoretical calculation of the anharmonic vibrational Raman and ROA spectra. Although it is not immediately obvious from the paper, the novelty rests in part with this being one of the first experimental studies of these spectra in such a wide frequency range, and secondly a further improvement of the VPT2 approach of some of the present authors in terms of its robustness and black-box nature. Although it can thus be argued that this perhaps is not fully novel, I find that this work, in particular its application to the reference molecule for chiroptical studies, methyloxirane, meets the high standards to be expected by papers published in the Journal of Physical Chemistry Letters.

In short, this paper points to possibly future exciting uses and opportunities offered by the combination of novel experimental advances in experimental Raman and ROA spectrometers in combination with advanced computational models.

The paper is overall well written and concise. However, in view of the comment above regarding my challenge with clearly identifying what was truly novel, I would recommend the authors to include a reference to Ref. 1 of the supplementary material (J.Phys.Chem. B 123, 2147 (2019)) at the end of the first paragraph of the introduction ("...advanced spectrometer") to make it clear these experimental advances are not directly reported in this paper. The authors may also consider whether to emphasise in the abstract or in the paper that this is one of the first applications of this new instrument.

On a very technical note, on manuscript page 4, I would recommend starting a new paragraph starting with "Recorded spectra...." as the focus switches from computational to experimental aspects.

On page 10, the sensitivity of resonance detection is critical for the reproducibility of the results reported. The authors note that they have improved this as well as the correction scheme. It would be good if the authors here could confirm that this entails the thresholds reported in the SI through a reference to the SI at this point, as well as add information on additional changes made if not described fully in the SI.

Author's Response to Peer Review Comments:

Pisa, 07 September 2022

Dear Editors,

Please find attached a revised version of the manuscript titled,

Anharmonic Vibrational Raman Optical Activity of Methyloxirane: Theory and Experiment Pushed to the Limits

By Qin Yang, Josef Kapitán, Petr Bouř and Julien Bloino

Manuscript ID: jz-2022-02320x

We would like to thank the reviewers for their comments and have amended our manuscript accordingly. We include below our reply to their comments.

REVIEWER 1

Manuscript is well written, provides all necessary details on computations and presents the results by very clear figures. I also appreciate that in addition to the main body of the text, which clearly present all most important aspects relevant for the broader audience, authors performed also detailed analysis of specific terms/effects affecting the final simulated spectra, which are very interesting for researchers working in the field. I only noticed that the text Harm/Anharm is reversed in the legend to the figure S7 (color code is OK).

Reply: We thank the reviewer for the comments. The legend has been corrected; we apologize for that.

REVIEWER: 2

1) The authors mention that the solvent effects have been taken into account via PCM. However, the use of the PCM normally reduces the achievable accuracy of gradients. I would like to see more discussion in that regard.

Reply: We thank the reviewer for the comments and suggestions. Although earlier versions of PCM did potentially affect the accuracy of numerical gradients and higher derivatives, we did not observe such problems. As recommended, a short discussion about this has been added to the text, together with comments on how this can be checked.

2) In the ESI, the authors correctly point out that different levels of theory can be reasonably combined only when the Duschinsky rotation and the shift vector are not large. It would be nice to have a table with characteristic parameters of the Duschinsky transformation between different levels of theory employed (and, perhaps, some related discussion).

*Reply: A graphical representation of the Duschinsky matrix (**J**) and shift vector (**K**) was added in the supplementary information (Fig. S9). More details were also added to the computational details.*

3) The findings seem to demonstrate that anharmonic corrections are not of very high importance to the fundamentals (except for C-H stretching, naturally) where simple scaling might suffice. Much more interesting is the possibility to explore the combination/overtone fingerprints. While the figures reveal apparent qualitative agreement between the experiment and theory, one would wonder, how critically would the computational results depend on the variations in the anharmonicity parameters? How important is their accuracy?

Reply: To highlight the improvements on the low-frequency fundamentals, Figure 2 with the regions dominated by fundamentals was magnified. The greater level of details now visible shows other benefits from the anharmonic corrections on the band-shape, beyond a simple shift of the bands. The gain over the use of scaling factors can also be noted from Figure S7 and Table S2.

Regarding the stability of the results, the harmonic part indeed represents a major contribution to the total energy, while the anharmonic contribution is smaller. This property is at the basis of hybrid schemes like the one used here. However, the choice of the "lower" level of theory is crucial to reach a high level of accuracy, as what is desired here. The overall sensitivity of the anharmonic calculation can be also estimated from Figure S2, where different DFT levels are compared. The frequency variation is smaller than 5 cm-1. The effect of the anharmonicity on the chiroptical intensities has been discussed, for example, in Phys. Chem. Chem. Phys. 2019, 21, 6582. The intensity accuracy is quite difficult to estimate and depends on the character of each bands. For our data and strongest fundamental/anharmonic bands, we may estimate the typical error as 10-20%.

REVIEWER: 3

The paper is overall well written and concise. However, in view of the comment above regarding my challenge with clearly identifying what was truly novel, I would recommend the authors to include a reference to Ref. 1 of the supplementary material (J.Phys.Chem. B 123, 2147 (2019)) at the end of the first paragraph of the introduction ("...advanced spectrometer") to make it clear these experimental advances are not directly reported in this paper. The authors may also consider whether to emphasise in the abstract or in the paper that this is one of the first applications of this new instrument.

Reply: We thank the reviewer for the comments. The reference was added, and the novelty of the experimental data was better explained in the first paragraph.

On a very technical note, on manuscript page 4, I would recommend starting a new paragraph starting with "Recorded spectra...." as the focus switches from computational to experimental aspects.

Reply: To keep the discussion more consistent, we moved the reference to Figure 1 earlier in the paper, and put the discussion of the PCM model in a dedicated paragraph.

On page 10, the sensitivity of resonance detection is critical for the reproducibility of the results reported. The authors note that they have improved this as well as the correction scheme. It would be good if the authors here could confirm that this entails the thresholds reported in the SI through a reference to the SI at this point, as well as add information on additional changes made if not described fully in the SI.

Reply: The sentence in the conclusions has been clarified, highlighting that the criteria had been tested and validated. Details on the tests have been added in the supplementary information (Figure S10).

Best regards,

Prof. Julien Bloino, PhD
Scuola Normale Superiore
Piazza dei Cavalieri, 7
I-56126 Pisa - Italy
tel: +39 050 509676
fax: +39 050 563513
email: julien.bloino@sns.it
